# Integrated single-cell and bulk RNA sequencing analysis identified pyroptosis-related signature for diagnosis and prognosis in osteoarthritis

**DOI:** 10.1038/s41598-023-44724-0

**Published:** 2023-10-18

**Authors:** Yanzhong Chen, Yaonan Zhang, Yongwei Ge, Hong Ren

**Affiliations:** 1 School of Sport Science, Beijing Sport University, Beijing, 100084 China; 2 Key Laboratory of Physical Fitness and Exercise, Ministry of Education, Beijing Sport University, Beijing, 10084 China; 3 Department of Orthopedics, Beijing Hospital, Beijing, 10000 China

**Keywords:** Computational biology and bioinformatics, Immunology

## Abstract

Osteoarthritis (OA), a degenerative disease of the joints, has one of the highest disability rates worldwide. This study investigates the role of pyroptosis-related genes in osteoarthritis and their expression in different chondrocyte subtypes at the individual cell level. Using OA-related datasets for single-cell RNA sequencing and RNA-seq, the study identified PRDEGs and DEGs and conducted Cox regression analysis to identify independent prognostic factors for OA. CASP6, NOD1, and PYCARD were found to be prognostic factors. Combined Weighted Gene Correlation Network Analysis with PPI network, a total of 15 hub genes related to pyroptosis were involved in the notch and oxidative phosphorylation pathways, which could serve as biomarkers for the diagnosis and prognosis of OA patients. The study also explored the heterogeneity of chondrocytes between OA and normal samples, identifying 19 single-cell subpopulation marker genes that were significantly different among 7 chondrocyte cell clusters. AGT, CTSD, CYBC, and THYS1 were expressed differentially among different cell subpopulations, which were associated with cartilage development and metabolism. These findings provide valuable insights into the molecular mechanisms underlying OA and could facilitate the development of new therapeutic strategies for this debilitating disease.

## Introduction

Osteoarthritis (OA) is a degenerative disease characterized by joint and muscle decline, synovial inflammation, and bone and cartilage decomposition. It is a prevalent condition among the elderly and a leading cause of disability worldwide, imposing a significant health and economic burden on individuals and society^1,2^. While the exact cause of OA remains unclear, it has been associated with age, obesity, inflammation, trauma, and genetics. Current treatments, including medication, non-pharmacological approaches, and surgery, have limited efficacy and can have adverse side effects^3,4^. Mesenchymal stem cell therapy has shown promise in promoting cartilage tissue repair and reducing inflammation, but more evidence is needed to support its use as a primary treatment option^5^.

Pyroptosis, a form of active cell death triggered by endogenous or exogenous stimuli, has been implicated in the pathophysiology of OA^6^. Mechanisms of pyroptosis involve mitochondrial dysfunction, inflammatory responses, and oxidative stress. The elevated levels of oxidative stress and expression of pyroptosis-related proteins found in joint tissues of OA patients further suggest the significance of pyroptosis in the pathogenesis of OA^7–10^.

To gain insight into the pathogenesis of OA related to pyroptosis, a bioinformatics analysis was performed using available RNA-seq data for OA. Our research aims to comprehensively understand the molecular status of osteoarthritis (OA) and identify key genes and pathways associated with cell apoptosis in this disease. To achieve this goal, we analyzed a large amount of transcriptomic data from both OA and control synovial tissues. Additionally, we also analyzed single-cell RNA sequencing (scRNA-seq) data from chondrocytes (cartilage cells). Differential analysis was conducted to identify differentially expressed genes (DEGs) between OA samples with high and low immune estimates. Pyroptosis-related differentially expressed genes (PRDEGs) were identified using the Mann–Whitney U test based on obtained pyroptosis-related genes (PRGs). Weighted Gene Correlation Network Analysis (WGCNA) was then applied to screen for pyroptosis-related modules, and Pearson correlation analysis was used to screen module genes. Key genes were obtained by intersecting DEGs and module genes, and a protein–protein interaction (PPI) network was constructed to identify hub genes. GO/KEGG function enrichment analysis, Gene set enrichment analysis (GSEA), and Gene set variation analysis (GSVA) were conducted based on hub genes, and mRNA-miRNA, mRNA-TF, and mRNA-drug networks of hub genes were constructed. Single-cell RNA sequencing (scRNA-seq) was used to reveal different chondrocyte subtypes and compare gene expression differences in different cell subpopulations. Immune infiltration analysis was performed to obtain immune cells with different levels of infiltration between the High-Pyroptosis score group and the Low-Pyroptosis score group.

This bioinformatics analysis provides insights into the pathogenesis of OA related to pyroptosis and identifies potential prognostic indicators. It also explores cell heterogeneity and expression differences of hub genes in different OA chondrocyte subpopulations, providing a new perspective on the management of OA.

## Methods

### Data collection and processing

We obtained the gene expression profiling microarray GSE12021^11^, GSE55235^12^,GSE82107^13^ and GSE89408^14^ of osteoarthritis (OA) from the Gene Expression Omnibus (GEO, https://www.ncbi.nlm.nih.gov/geo/) database using “GEOquery” R package^15^. Thereinto, the GSE12021 contains 19 samples, including 10 OA samples and 9 control samples. The GSE55235 consists of 20 samples, including 10 OA samples and 10 control samples. GSE82107 contains 17 samples, including 10 OA samples and 7 control samples. The GSE89408 consists of 49 samples, including 22 OA samples and 27 control samples. It is important to emphasize that we excluded all RA (rheumatoid arthritis) samples because our research primarily focuses on the pathophysiological processes of osteoarthritis, rather than conducting a comparative study involving various types of arthritis. Although both RA and osteoarthritis are arthritic conditions, they have significantly different pathogenic mechanisms and pathological processes. To maintain the clarity and specificity of our research, we chose to include only samples from osteoarthritis patients and excluded samples from other types of arthritis. The details of the 4 GEO dataset samples are shown in Table 1. We first merged the datasets GSE12021, GSE55235, GSE82107 and GSE89408, then removed the batch effect using the “sva” R package^16^. “Sva” (Surrogate Variable Analysis) is a commonly used R software package in gene expression data analysis. It is a method employed to detect and adjust for potential batch effects, aiming to enhance the reliability and accuracy of differential expression analysis and expression level clustering in various research studies. Next, we applied the “limma” R package^17^ to standardize the merged datasets to obtain an OA dataset. The “limma” is a widely used R software package in gene expression data analysis. It is a linear modeling method designed for differential expression analysis and is applicable for both microarray and RNA sequencing data. Then Principal Component Analysis (PCA) was performed on the expression matrix of the datasets before and after the removal of batch effect to verify the effect of removing the batch effect. PCA is a method of data dimensionality reduction. It extracts feature vectors (components) of data from high-dimensional data, converts them into low-dimensional data, and uses two-dimensional or three-dimensional graphs to display these features.

**Table 1:** Dataset details.

GEO ID	Species	OA sample	Control sample	Organization source	GPL ID
GSE12021	Homo sapiens	10	9	synovial membrane	GPL96[HG-U133A] Affymetrix Human Genome U133A Array
GSE55235	Homo sapiens	10	10	synovial membrane	GPL96[HG-U133A] Affymetrix Human Genome U133A Array
GSE82107	Homo sapiens	10	7	synovial membrane	GPL96[HG-U133A] Affymetrix Human Genome U133A Array
GSE89408	Homo sapiens	22	27	synovial membrane	GPL570[HG-U133_Plus_2] Affymetrix Human Genome U133 Plus 2.0 Array

This scRNA-seq data set, GSE169454, was retrieved from the GEO database and was generated using the GPL555 Illumina HiSeq 2500 platform (Homo sapiens). The samples were collected from 4 patients with knee osteoarthritis and 3 normal subjects. We selected 5 single cell samples of chondrocytes, among which 3 were normal (Normal1: GSM5203389, Normal2: GSM5203390, Normal3: GSM5203391) and 2 were OA (OA1: GSM5203392, OA2: GSM5203393).

Seurat is a widely used R software package in the analysis of single-cell RNA sequencing (scRNA-seq) data. It is a highly flexible and powerful tool for processing, visualizing, and analyzing gene expression data at the single-cell level. For single-cell data processing, we used the “Seurat” R package^18^ to create five single-cell data as Seurat objects, and calculated the percentage of mitochondria in each cell through the "PercentageFeatureSet" function of the “Seurat” package. It is generally believed that when a cell has a high proportion of mitochondrial genes, it might be in a state of apoptosis or lysis. Therefore, for each cell, the proportion of mitochondrial genes was computed, and cells having a mitochondrial fraction more than 5% were filtered away. Due to the fact that cells with fewer than 200 genes were found to be of poor quality or to be empty droplets, and that cell duplication or multiplication might result in an excessively large gene count, we also filtered out cells with feature < 500. In addition, we filtered cells with total UMI > 20,000. Subsequently, we obtained 22,839 chondrocytes from patients with osteoarthritis and 6,360 normal chondrocytes.

After completing the quality control procedure, OA samples (OA1, OA2) and normal samples (Normal1, Normal2, Normal3) were integrated respectively, and batch effects were removed using the CCA method in Seurat with the functions named the "FindIntegrationAnchors" and "IntegrateData". For Seurat objects, utilising the most differentially expressed genes, we did linear dimensional decrease using principal components (PC) calculation^19^. Then, we used "FindNeighbors" and "FindClusters" functions of Seurat to group cells into clusters with the optimum cluster size to identify the cell type. Then to compress the data gathered in the chosen important main components to two dimensions, we employed tSNE (t-Distributed Neighbor Embedding), realizing a visual graph-based clustering of cells. In addition, 33 pyroptosis-related genes (PRGs) were obtained based on previous studies. In addition, 33 pyroptosis-related genes (PRGs) were obtained based on previous studies^20^.

### Cell annotation

Cell annotation is the process of associating and labeling each cell in single-cell sequencing data with its corresponding cell type, which helps researchers identify the cell types of individual cells and explore transcriptional differences in different physiological states and diseases. For osteoarthritis samples, 14 clusters were visualized using tSNE. For the normal samples, a total of 10 clusters were visualized using tSNE, and seven distinct chondrocyte subtypes were revealed by artificial annotation of cell type marker genes. The marker genes of homeostatic chondrocytes (HomC) contain MMP3, FOSB and JUN. The marker genes of hypertrophic chondrocytes (HTC) contain COL10A1, IBSP and JUN. The marker genes of prehypertrophic chondrocytes (preHTC) include COL10A1, IBSP, COL2A1 and TGFBI. The marker genes of regulatory chondrocytes (RegC) include CHI3L1 and CHI3L2. The marker genes of fibrochondrocytes (FC) contain COL1A1, COL1A2, S100A4, PRG4 and TMSB4X. The marker genes of reparative chondrocytes (RepC) include COL2A1, CILP, COL3A1 and COMP. The marker genes of preFC contain IL11, COL2A1, CILP and OGN^18,19,21–23^. To understand the expression patterns of diagnostic markers in patients with osteoarthritis, we compared gene expression in different cell groups.

### Immunologic estimate

The level of tumor microenvironment cell, immune cell, and stromal cell invasion have significant influence on the prognosis. To clarify the influence of genes involved in immunity and stromal cells on prognosis, the “ESTIMATE” R package^24^ could leverage the uniqueness of the tumor sample's transcriptional pattern to deduce the composition of tumour cells and the various invading normal cells. We used the “ESTIMATE” to estimate the immune activity of samples by using the expression profile matrix data of the OA dataset. Then ESTIMAE score, Immune score and Stromal score of the characteristics of the expression matrix were calculated based on the ESTIMATE algorithm to quantify the immune and stromalscore components of the sample. Next, we separated OA samples into two categories, one with a high ImmuneScore and another with a low ImmuneScore, based on the midpoint of the distribution. The “limma” R package was applied to conduct differential expression analysis, and differentially expressed genes (DEGs) were discovered in distinct groups. We chose | logFC |> 0.5 and *p* value < 0.05 as the standard screened genes for further study. Thereinto, genes with logFC > 0.5 and *p* value < 0.05 were up-regulated genes and those with logFC < − 0.5 and *p* value < 0.05 were down-regulated genes. The consequences were presented as the volcano map drawn by “ggplot2” R package^25^ and the heatmap drawn by “pheatmap” R package^26^.

### Calculation of pyroptosis score based on OA dataset

Firstly, the Mann–Whitney U test was used to evaluate the difference between OA and control samples with regards to PRG expression (WilCoxon rank sum test).With a P-value less than 0.05, the results were considered significant, and pyroptosis-related differentially expressed genes (PRDEGs) were discriminated.

The single-sample gene-set enrichment analysis (ssGSEA) algorithm could determine how many times each gene appears in the database samples. The “GSVA” R package^27^ was applied to count the Pyroptosis score of each sample in the OA group by the ssGSEA algorithm on account of the PRDEGs expression matrix of each sample in the OA dataset. Based on the median Pyroptosis value, we separated the OA samples into two groups: High-Pyroptosis and Low-Pyroptosis.

### Enrichment analysis between the High-Pyroptosis score group and the Low-Pyroptosis score group

For the goal of comprehending the distinctions between the two groups' biological systems, GSEA^28^ was performed using gene expression profile data of OA patients. GSEA is a computer tool that permits the evaluation of whether a particular gene set demonstrates numerically substantial improvements between two biological states. The “clusterProfiler” R package^29^ was used to perform GSEA, with a P-Value of less than 0.05 indicating considerably enhanced data.

GSVA is a method of gene set enrichment analysis for unduplicated samples. The score of the corresponding gene set could be obtained for each sample through GSVA, and the differentially expression analysis could be performed for pathways of each sample to obtain differentially expressed pathways between groups. We downloaded the reference gene set from MSigDB database^30^ (https://www.gsea-msigdb.org/gsea/msigdb/). Finally, the "GSVA" R program was utilized to find commonalities between samples from the high-risk and low-risk groups. Also, we used “limma” R package to conduct differentially expression analysis on scores of various pathways between two groups. The parameter is set to *p* value < 0.05 and | logFC |> 0, which is displayed by heatmap.

### Identification of key genes

The goals of WGCNA^31^ are to identify modules of co-expressed genes, study how genetic network correlate with phenotypes, and dissect key genes within these networks. The pickSoftTreshold method ultimately determined a value of 12 to be the best possible soft threshold. Subsequently, using this flexible limit, we formed a topological matrix, clustered the data hierarchically, and built a scale-free network. With 50 as the minimum number of modules, gene modules were actively slashed and identified, and Eigengenes were computed. Correlation between modules was built on account of module Eigengenes, hierarchical clustering was conducted, and modules with correlation greater than 0.5 were combined again, and finally 5 modules were gained. The association between module pyroptosis scores was analysed using Pearson's method, and candidate module genes (MEGs) were eliminated by this process (MEGs). Subsequently, we took the intersection of DEGs and MEGs to obtain genes as key genes in this study.

### Construction of PPI network and identification of hub genes

STRING (Search Tool for the Retrieval of Interacting Genes/Proteins) is an online tool used to search and analyze the interactions between proteins and genes. It provides extensive information on protein interactions, helping researchers explore protein–protein interaction networks and functional regulatory mechanisms. PPI network predictions and network construction were performed using the online STRING (a search engine used to find interacting genes) tool^32^. The PPI network was seen using Cytoscape^33^, and hub genes were filtered using the cytoHubba^34^ extension.

### GO and KEGG enrichment analysis

Gene Ontology (GO) analysis has been utilized as a general approach for mass functional enrichment, including biological processes (BP), molecular functions (MF) and Cellular Component (CC)^35^. The Genomes, biological processes, illnesses, and medications material is stored in the Kyoto Encyclopedia of Genes and Genomes (KEGG)^36^. For both DEGs and hub genes, we performed GO annotation analysis and KEGG pathway enrichment analysis using the "clusterProfiler" R package. We arbitrarily chose a significance level of 0.05 for the FDR threshold. The top 8 outcomes of GO and the top 10 results of KEGG are shown in the bar chart and dot chart, respectively.

### Construction of mRNA-miRNA, mRNA-TF, mRNA-drugs interaction network 

ENCORI^37^ database (https: // Starbase.sysu.edu.cn/) is the 3.0 version of the starBase database. The ENCORI database provides multiple graphical user interfaces for discussing microRNA targets, including those between miRNA and ncRNA, miRNA and mRNA, ncRNA and RNA, RNA and RNA, and RBP and ncRNA or RBP and mRNA. These interactions are supported by data mined from CLIP-seq and degradation sequencing experiments. Predictions of miRNA target genes and their functions were made using the miRDB database^38^. In this study, miRNAs interacting with hub genes (mRNA) were forecasted in accordance with ENCORI and miRDB databases, and the mRNA-miRNA connection system was plotted using the intersection of the two databases.

CHIPBase database^39^ (https://rna.sysu.edu.cn/chipbase/) found a matrix of binding sites for hundreds of DNA motifs, and predicted millions of Transcription factor (Transcription factors, TF) and transcriptional regulatory relationships between genes from the DNA binding protein CHIP-seq data. hTFtarget database^40^ (http://bioinfo.life.hust.edu.cn/hTFtarget) refers to an integrated database of human TF and target regulation. Using the CHIPBase (version 3.0) and hTFtarget databases, we looked for transcription factors (TFS) that bind to hub genes.

Comparative Toxicogenomics Database (CTD)^41^ (http://ctdbase.org/) is based on an innovative digital ecosystem linking chemicals, genes, phenotypes, diseases and known toxicological information, which could be used to facilitate access to human health related information. With References ≥ 3 and Organisms ≥ 3 as screening criteria, we also use the CTD database to predict latent drugs or small molecule compounds interacting with hub genes, and then visualize the mRNA-miRNA, mRNA-TF, mRNA-drugs interaction network via Cytoscape.

### Immune infiltration analysis

In order to calculate the distribution and concentration of immune cells among the mixed cells, CIBERSORT^42^ used a deconvolution of the transcriptional expression matrix based on the idea of linear support vector regression. Using CIBERSORT, we integrated the OA dataset with the LM22 signature gene matrix. The output samples were filtered with *p* < 0.05, and the immune cell infiltration matrix was acquired. Histograms of the 22 different kinds of immune cells present for each sample were generated using the "ggplot2" R package. The correlation between immune cells were displayed as heatmap. Following that, we evaluated the immune cell ratings between the High and Low Pyroptosis groups. Thus, immune cells with various infiltration levels between the two groups were obtained.

### Statistical analysis

All of the calculations and statistical analyses in this study were performed in R (https://www.r-projec t.org/, version 4.1.2). In order to determine the statistical significance of differences between two sets of normally distributed data, the independent Student t test was utilised, whereas the Mann–Whitney U test (i.e., Wilcoxon rank sum test) was used to evaluate differences between non-normally distributed variables. All *p* values were calculated on a two-sample basis, and a *p* value less than 0.05 was considered statistically significant. Generalized linear model (glm) was used in the construction of mono—and multi-factor forest graph models, and corresponding nomogram was built using the “rms” R package^43^. If not specified specifically, in this research, the Spearman correlation analysis was used to get the correlation coefficients between the variables. P values were calculated on both sides of the equation, and values below 0.05 were considered significant statistically.

## Results

### Batch effect removal and differential gene expression analysis reveals immune dysregulation in osteoarthritis

**Figure 1:**
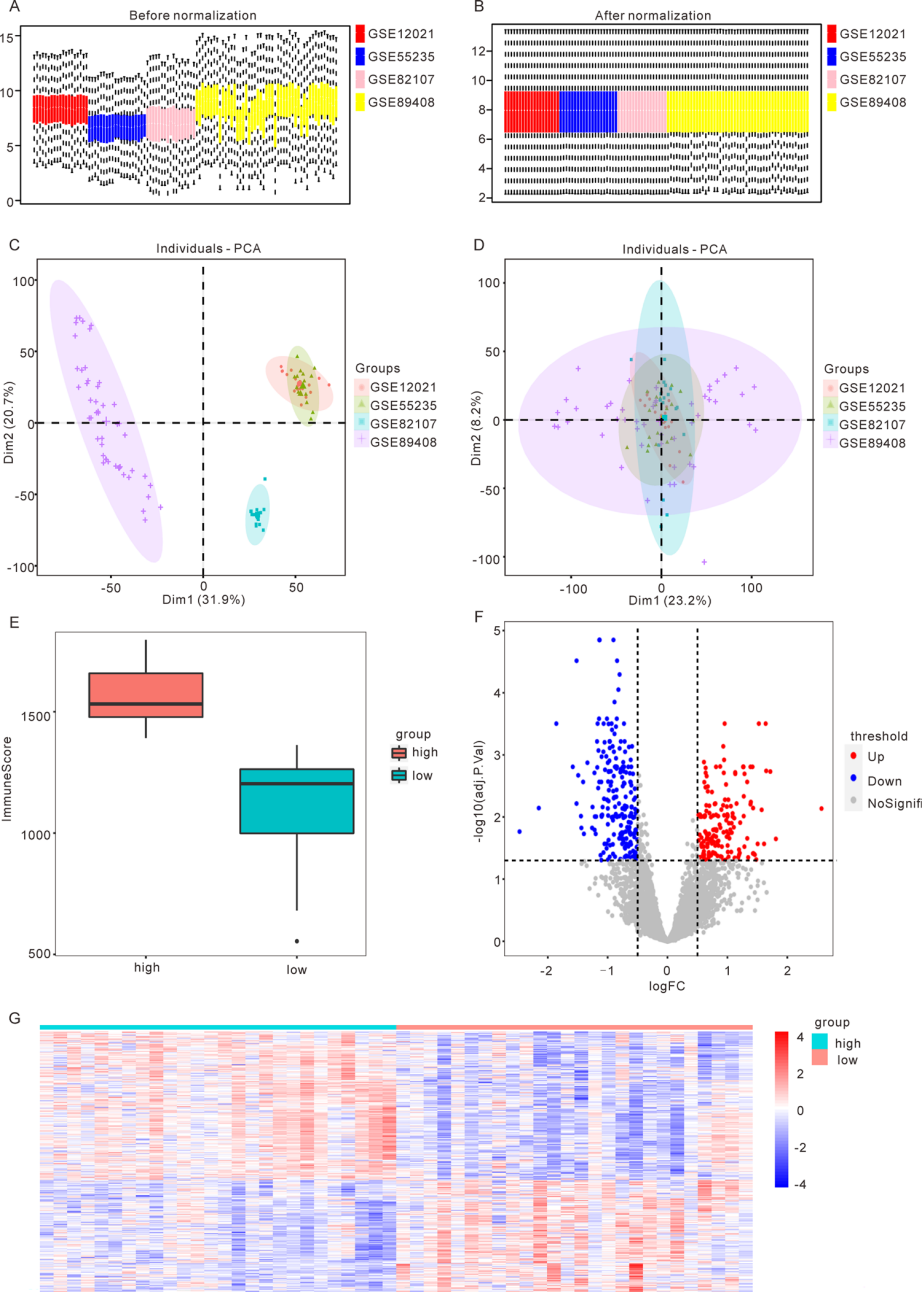
Identification of differentially expressed genes based on the merged osteoarthritis (OA) dataset. (**A**) boxplot of OA datasets before standardization. (**B**) boxplot of OA datasets after standardization. (**C**) PCA plot of OA datasets before batch effect removal. (**D**) PCA plot of OA datasets after batch effect removal. (**E**) Distribution of ImmuneScore in High ImmuneScore and Low ImmuneScore groups in OA samples. (**F**) The volcano map showed the results of differentially expression analysis, with 160 up-regulated genes and 212 down-regulated genes (red represents up-regulated expression, blue represents down-regulated expression). (**G**) The heatmap shows the expression of DEGs between the High ImmuneScore group and the Low ImmuneScore group. (OA: osteoarthritis).

Firstly, we combined four datasets related to osteoarthritis (OA): GSE12021, GSE55235, GSE82107, and GSE89408. We utilized the “sva” R packages to remove batch effects and the “limma” R package to standardize the combined dataset, resulting in the OA data set required for our analysis. This dataset contained 52 OA samples and 53 control samples (Fig. 1A,B.) To verify the effectiveness of the batch effect removal, we conducted principal component analysis (PCA) on the expression matrix of the data set before and after batch effect removal, based on the source of the samples (Fig. 1C,D). The results demonstrated that batch effects from samples from different sources in the OA data set were largely eliminated after the removal of batch effects.

To determine the tumor purity, we utilized the “ESTIMATE” R package, which infers the contents of tumor cells and different infiltrating normal cells by calculating immune and mechanical scores based on RNA-seq data. Using the “ESTIMATE” R package, we calculated the expression profile data of 52 sorted OA samples and obtained various immune and matrix scores for each sample. Based on the median ImmuneScore, we divided the samples into the High ImmuneScore group (n = 26) and the Low ImmuneScore group (n = 26) (Fig. 1E). To compare the differences between these groups, we used the “limma” R package to identify differentially expressed genes (DEGs), obtaining 160 up-regulated genes and 212 down-regulated genes (Fig. 1F). We then generated a heatmap to illustrate the expression patterns of the DEGs in the High ImmuneScore group and the Low ImmuneScore group, revealing significant differences in gene expression patterns between the two groups (Fig. 1G).

### Functional enrichment analysis reveals immune-related pathways and pyroptosis-associated genes in osteoarthritis

**Figure 2:**
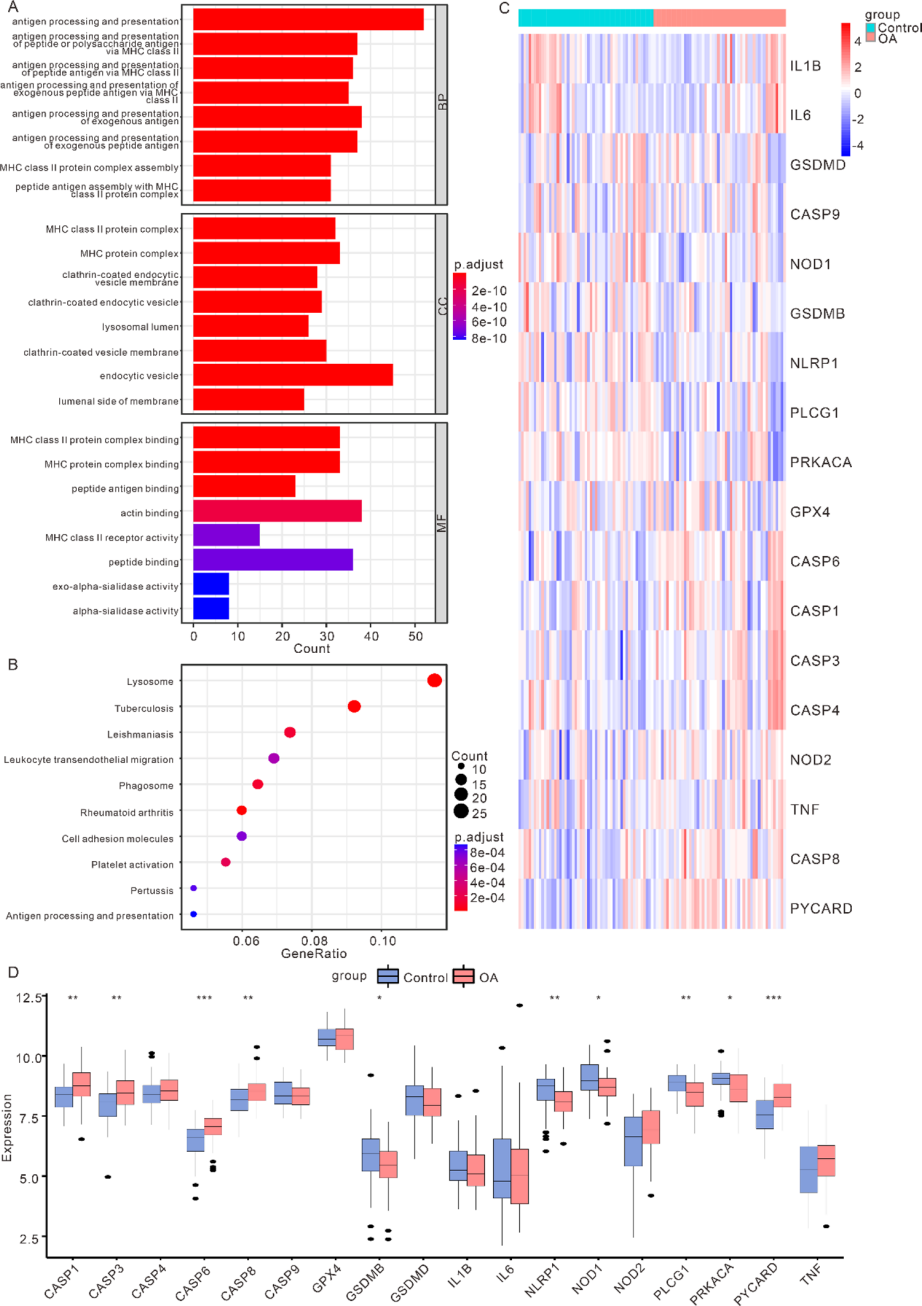
Panorama of cell apoptosis-related genes based on the merged osteoarthritis (OA) dataset. (**A**) Enrichment of DEGs in biological process (BP), cellular component (CC), molecular function (MF) in Gene Ontology (GO) enrichment analysis. (**B**) pathways of DEGs enrichment in the Kyoto Encyclopedia of Genes and Genomes (KEGG) pathway enrichment analysis. The larger the circle, the higher the enrichment level. (**C**) Expression heatmap of PRGs in OA datasets. Red represents high expression, while blue represents low expression. (**D**) Comparison chart of the differentially expression analysis of PRGs in OA datasets (*p < 0.05; **p < 0.01; ***p < 0.001). (PRGs: pyroptosis-related genes; OA: osteoarthritis).

GO and KEGG enrichment analyses were conducted on DEGs. The GO analysis demonstrated that the DEGs had relation to antigen processing and presentation of peptide or polysaccharide antigen via MHC class II, antigen processing and presentation of peptide antigen via MHC class II and other biological processes, MHC class II protein complex, MHC protein complex, clathrin-coated endocytic vesicle membrane and other cell components, as well as MHC class II protein complex binding, MHC protein complex binding, peptide antigen binding and other molecular function(Fig. 2A). The KEGG analysis demonstrated that the DEGs had relation to Lysosome, Tuberculosis, and Leishmaniasis pathways (Fig. 2B).

There were 33 PRGs obtained from previous study. And we interacted with the genes in the OA datasets. And then a total of 18 PRGs were obtained (CASP1, CASP3, CASP4, CASP6, CASP8, CASP9, GPX4, GSDMB, GSDMD, IL1B, IL6, NLRP1, NOD1, NOD2, PLCG1, PRKACA, PYCARD, TNF). The “heatmap” was shown using the “pheatmap” R package, which demonstrated the expression of PRGs in OA samples and control samples (Fig. 2C). In order to analyze the positions of PRGs on human chromosomes, we also used the “RCircos” R package to annotate the positions of 18 PRGs (Fig. 2D). As shown in Fig. 2D, these PRGs were mainly distributed on chromosomes 2, 4, 6, 7, 8, 11, 16, 17, 19 and 20. Finally, in order to identify PRDEGs, we performed differentially expression analysis of the expression of 18 PRGs between OA samples and control samples by Wilcoxon signed rank test, which uncovered that CASP1, CASP3, CASP6, CASP8, GSDMB, NLRP1, NOD1, and the expression difference of 18 PRGS between OA samples and control samples. The outcomes demonstrated that the differentially expression of CASP1, CASP3, CASP6, CASP8, GSDMB, NLRP1, NOD1, PLCG1, PRKACA, PYCARD and other 10 genes in different samples were scientifically meaningful (Fig. 2E, *p* < 0.05).

### Prognostic significance of pyroptosis-related genes in osteoarthritis

**Figure 3:**
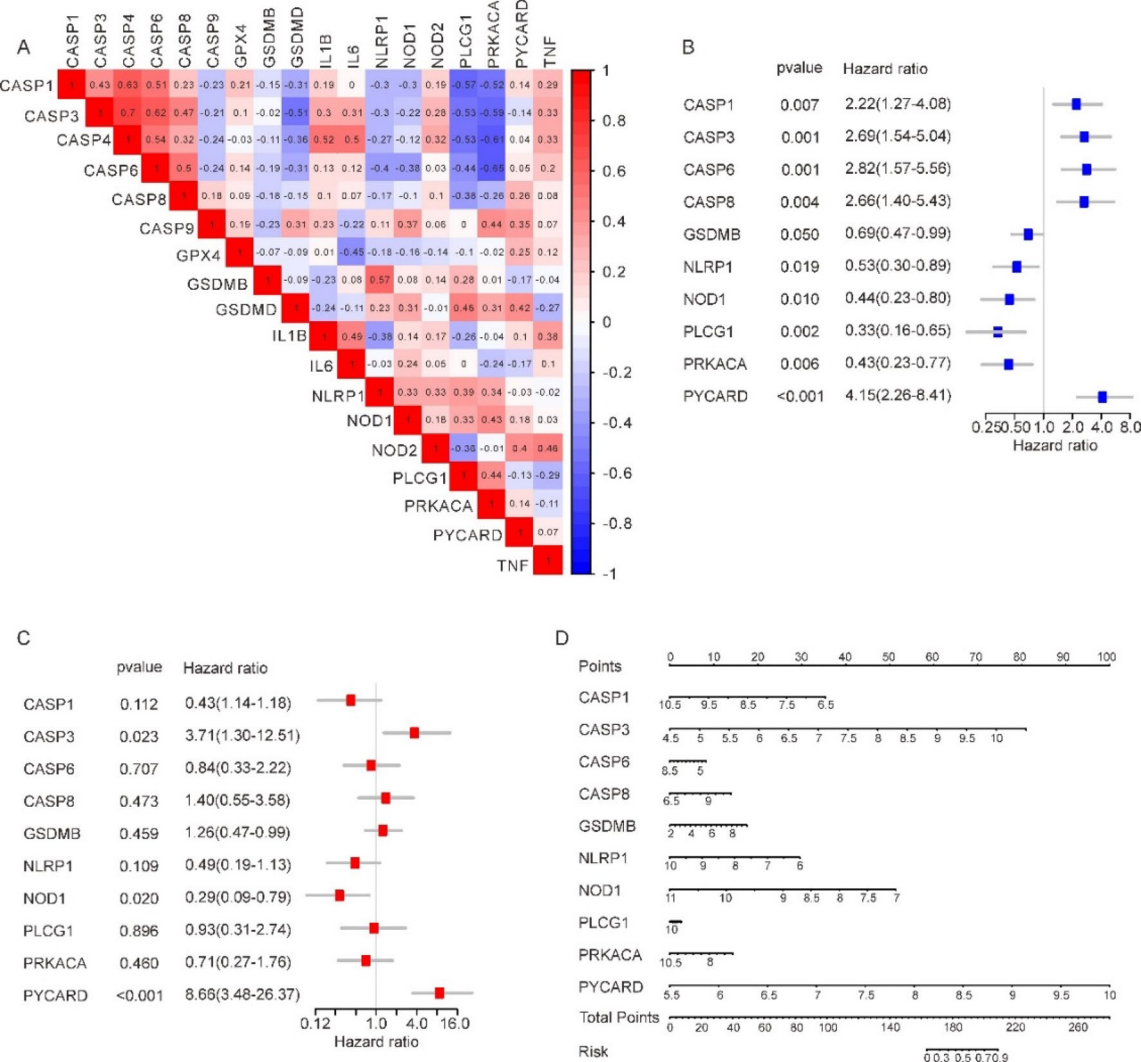
Forest map model built in OA datasets based on PRDEGs. (**A**) Correlation heatmap between 18 PRGs. Red represents positive correlation, while blue-purple represents negative correlation. (**B**) The univariate COX analysis results of PRDEGs. (**C**) The multivariate COX analysis results of PRDEG. (D) nomogram of PRDEG. (PRGs: pyroptosis-related genes; PRDEGs: pyroptosis related differentially expressed genes).

Firstly, we analysed the connection between PRGs using Pearson’s method, and the results were shown in Fig. 3A. The positive correlation between CASP1 and CASP4 was the largest (r = 0.63, *p* = 4.47E−07), and the negative correlation between PRKACA and CASP6 was the largest (r = − 0.65, *p* = 2.32E−07).

The OA datasets were analysed using univariate and multivariate Cox risk regression to determine whether PRDEGs were an independent prognostic factor. Univariate analysis showed that CASP1, CASP6, CASP8, NLPR1, NOD1, PLCG1, PRKACA, and PYCARD could be independent prognostic factors (Fig. 3B), while multivariate analysis showed that only CASP6, NOD1 and PYCARD could be considered as independent prognostic factors (Fig. 3C). Subsequently, in order to further evaluate the contribution of each factor in the multivariate Cox risk regression analysis to OA events, the nomogram was constructed in accordance with the consequences of the multivariate Cox risk regression analysis, and scores were assigned to each PRDEGs (Fig. 3D). The analysis revealed PYCARD had the highest score, which was also compatible with the consequences of former analysis. Multivariate and univariate Cox risk regression analysis found that the PYCARD gene had the greatest Hazard ratio.

### Identification of hub genes and co-expression modules associated with pyroptosis in osteoarthritis

**Figure 4:**
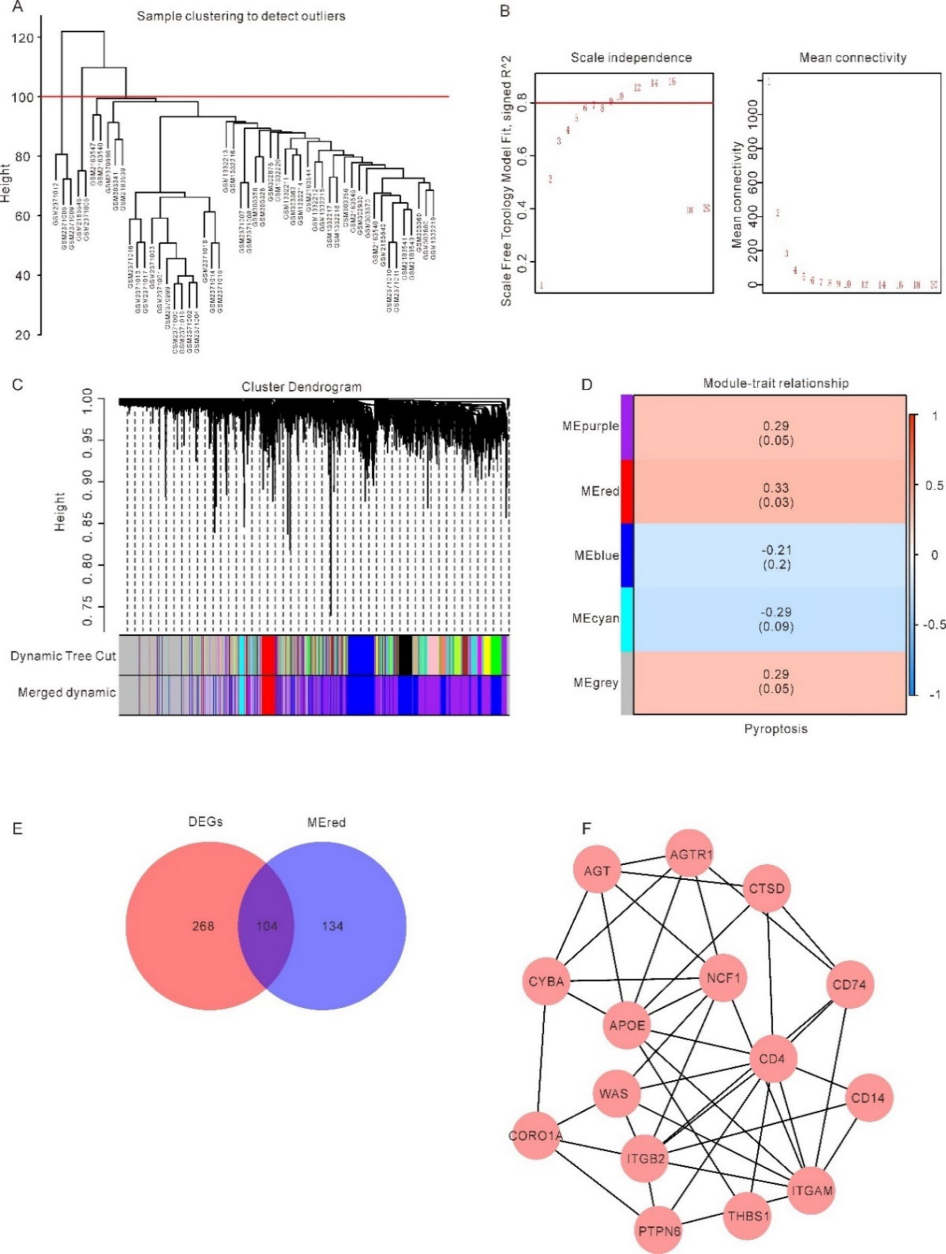
Identification of hub genes based on the integrated OA datasets. (**A**) eliminate outlier samples through cut height. (**B**) determination of soft threshold of optimal soft power. (**C**) the aggregation process of module genes. (**D**) correlation between module genes and Pyroptosis score. (**E**) A total of 238 modular genes were intersected with 372 DEGs. (**F**) The top 15 genes of MCC selected as hub genes through CytoHubba and the edges represent the correlations between them.

Firstly, on account of the 10 PRDEGs, each OA sample's level of pyroptosis was quantified by calculating its Pyroptosis Score with the help of the ssGSEA Algorithm.We then conducted WGCNA in 52 OA samples to screen for co-expression modules. In the process of WGCNA, 5 outlier samples were eliminated by setting cut height (Fig. 4A). The scatter plot enabled us to choose 12 as the optimal soft threshold and carried out follow-up research (Fig. 4B). We then merged the modules with a cut height less than 0.5 and grouped the genes in the OA samples into 5 modules, namely MEpurple, MEred, MEblue, MEcyan, and MEgrey (Fig. 4C). Finally, we assessed the correlation between each module and the Pyroptosis score of each OA patient through Pearson correlation analysis (Fig. 4D). We selected the MEred module with the highest correlation coefficient for further analysis, which contained 238 genes (Supplementary Table S1).

Subsequently, we intersected 238 modular genes with 372 DEGs, and 104 key genes were obtained (Fig. 4E and Supplementary Table S2). Finally, STRING database was applied to establish PPI network among 104 key genes. The interactions between proteins were imported into Cytoscape software and the MCC algorithm was used to screen the top 15 genes (WAS, AGT, CTSD, APOE, CYBA, NCF1, ITGAM, AGTR1, CD74, CORO1A, ITGB2, PTPN6, THBS1, CD4, CD14) from the PPI network through the CytoHubba plugin of Cytoscape. as hub genes (Fig. 4F).

### Integrated analysis of mRNA-miRNA, mRNA-TF, and mRNA-Drugs interactions in osteoarthritis

Using the ENCORI and miRDB databases, connections between 15 hub genes and interacting microRNAs were predicted (WAS, AGT, CTSD, APOE, CYBA, NCF1, ITGAM, AGTR1, CD74, CORO1A, ITGB2, PTPN6, THBS1, CD4, CD14), and Cytoscape software was used to construct mRNA-miRNA interaction network for visualization (Supplementary Fig. S1). The red oval block represented mRNA and the blue oval block represented miRNA in the mRNA-miRNA interaction network. In our study, the mRNA-miRNA interaction network is composed of 7 hub genes (WAS, AGT, CTSD, AGTR1, CD74, THBS1, CD4) and 48 miRNA molecules.

We searched the CHIPBase database (version 3.0) and the hTFtarget database for Transcription factors (TF) which bind hub genes. Interactions found in both databases were downloaded with 15 hub genes, finally, 13 hub genes (AGT, AGTR1, APOE, CD14, CD4, CD74, CORO1A, CTSD, CYBA, ITGB2, PTPN6, THBS1, WAS) and 132 transcription factors (TFS) were obtained, which were visualized by Cytoscape software. mRNA is represented by the red oval block and the transcription factor by the blue oval block in the mRNA-TF interaction network (TF) (Supplementary Fig. S2).

15 hub genes have their pharmacological or chemical compound potentials investigated using the CTD database. We identified 14 hub genes from the CTD database (AGT, AGTR1, APOE, CD14, CD4, CD74, CORO1A, CTSD, CYBA, ITGAM, ITGB2, NCF1, PTPN6, THBS1) corresponding to 70 potential drugs or molecular compounds. The mRNA is represented by a red oval block and the drugs as a blue oval block in the mRNA-drugs interaction diagram (Supplementary Fig. S3).

### Functional enrichment analysis of hub genes and pyroptosis-related pathways in osteoarthritis

**Figure 5:**
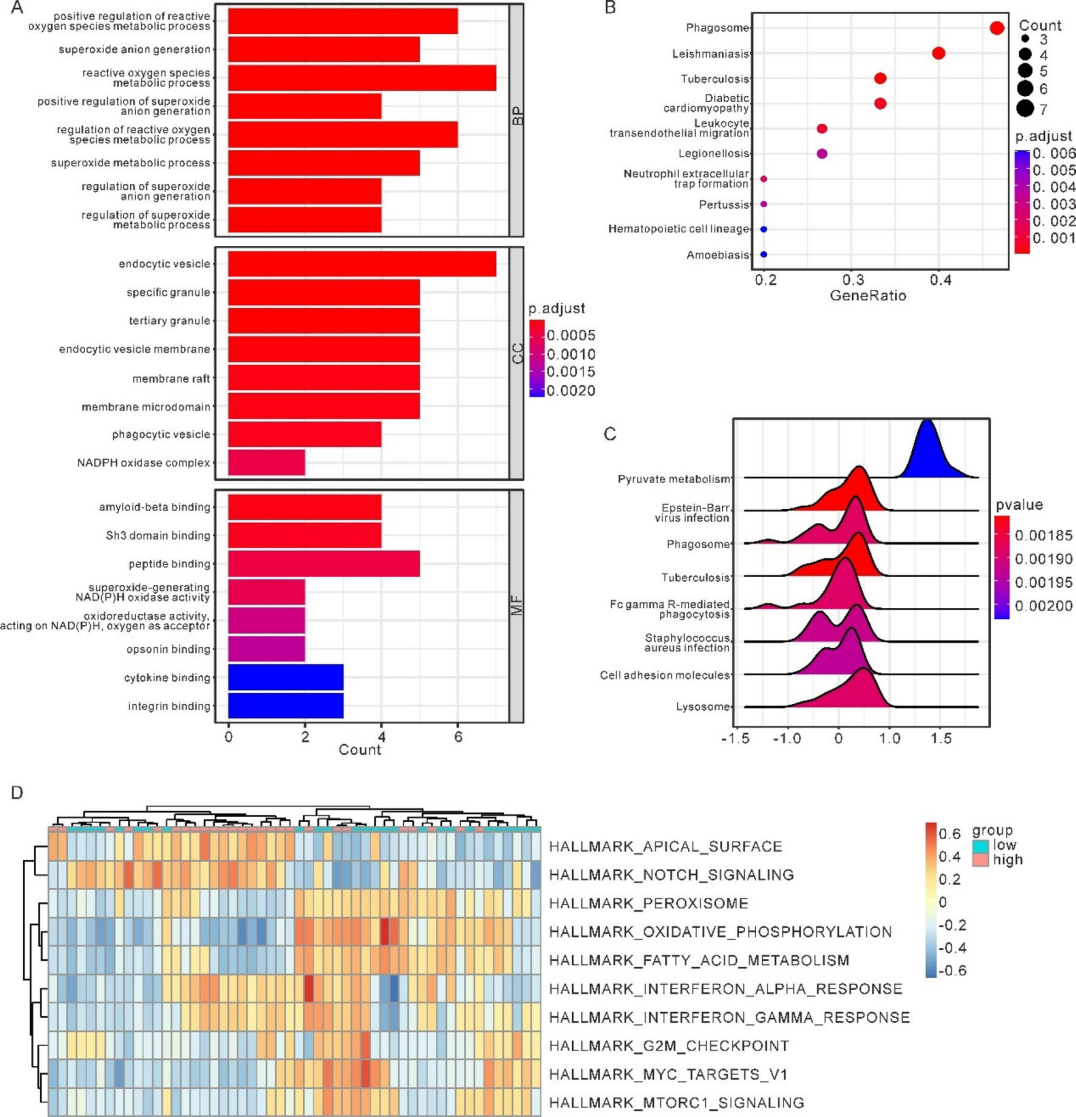
GSEA and GSVA between High-Pyroptosis score group and Low-Pyroptosis score group on the integrated OA datasets. (**A**) Enrichment of hub genes in biological process (BP), cellular component (CC), molecular function (MF) in Gene Ontology (GO) enrichment analysis. (**B**) The pathways of hub genes in the Kyoto Encyclopedia of Genes and Genomes (KEGG) pathway enrichment analysis. (**C**) The top 8 pathways with the lowest P value in GSEA. (**D**) Complex numerical heatmap of inter-group GSVA results of high and low Pyroptosis score in OA datasets. (OA: osteoarthritis; GSEA: Gene set enrichment analysis; GSVA: Gene Set Variation Analysis).

GO and KEGG enrichment analyses was conducted upon the 15 hub genes. GO analysis indicated hub genes were connected with positive regulation of reactive oxygen species metabolic process, superoxide anion generation, reactive oxygen species metabolic process and other biological process, endocytic vesicle, specific granule, tertiary granule and other cell component, as well as amyloid-beta binding, SH3 domain binding, peptide binding and other molecular function (Fig. 5A). KEGG analysis suggested hub genes were enriched in Phagosome, Leishmaniasis, and Tuberculosis pathways (Fig. 5B).

As previously stated, the ssGSEA technique, which uses 10 PRDEGs to quantify pyroptosis, was used to calculate a score for each OA patient. In addition, two distinct cohorts of OA samples were analysed, one with a high median Pyroptosis score and the other with a low median Pyroptosis score. We then compared gene expression between the high and low groups. The GSEA was conducted according to the log2FoldChange. Figure 5C shows the first 8 enrichment results with the lowest P-value, including Tuberculosis, Epstein-Barr virus infection, Phagosome, Lysosome, Fc gamma R-mediated phagocytosis, Staphylococcus aureus infection, Cell adhesion molecules, Pyruvate metabolism pathway. Supplementary Fig. S4 A-H shows the specific enrichment of related pathways. In order to explore the differences of h.all.v 7.5.1. symbols reference gene set pathways between the High-Pyroptosis score group and the Low-Pyroptosis score group in OA datasets, we performed GSVA between the two groups. The analysis results show that apical surface, interferon alpha response, MYC targets V1, mtorC1 signaling, oxidative phosphorylation, peroxisome, interferon gamma response, notch signaling, fatty acid metabolism, G2M checkpoint, a total of 10 h.all.v 7.5.1.symbols reference gene set pathways exhibited significant differences between the two groups in the OA datasets (*p* < 0.05). Thereinto, the enrichment scores of apical surface, interferon alpha response, notch signaling, interferon gamma response channels in the High-Pyroptosis score group were significantly higher than those in the Low-Pyroptosis score group. While the enrichment scores of G2M checkpoint, fatty acid metabolism, peroxisome, mtorC1 signaling, MYC targets V1 and oxidative phosphorylation in the High-Pyroptosis score group were significantly lower than those in the Low-Pyroptosis score group. Based on the results of GSVA, we looked at how differently 10 pathways were expressed between the two groups in the OA datasets, and “pheatmap” R package was employed to plot heatmap, which shows the specific various analysis results (Fig. 5D). As Fig. 5D shown, there were significant differences in the expression of Pyroptosis score between the OA and the pyroptosis score groups in the 10 pathways obtained by GSVA.

### Immune cell infiltration analysis and correlation with hub genes in osteoarthritis

**Figure 6:**
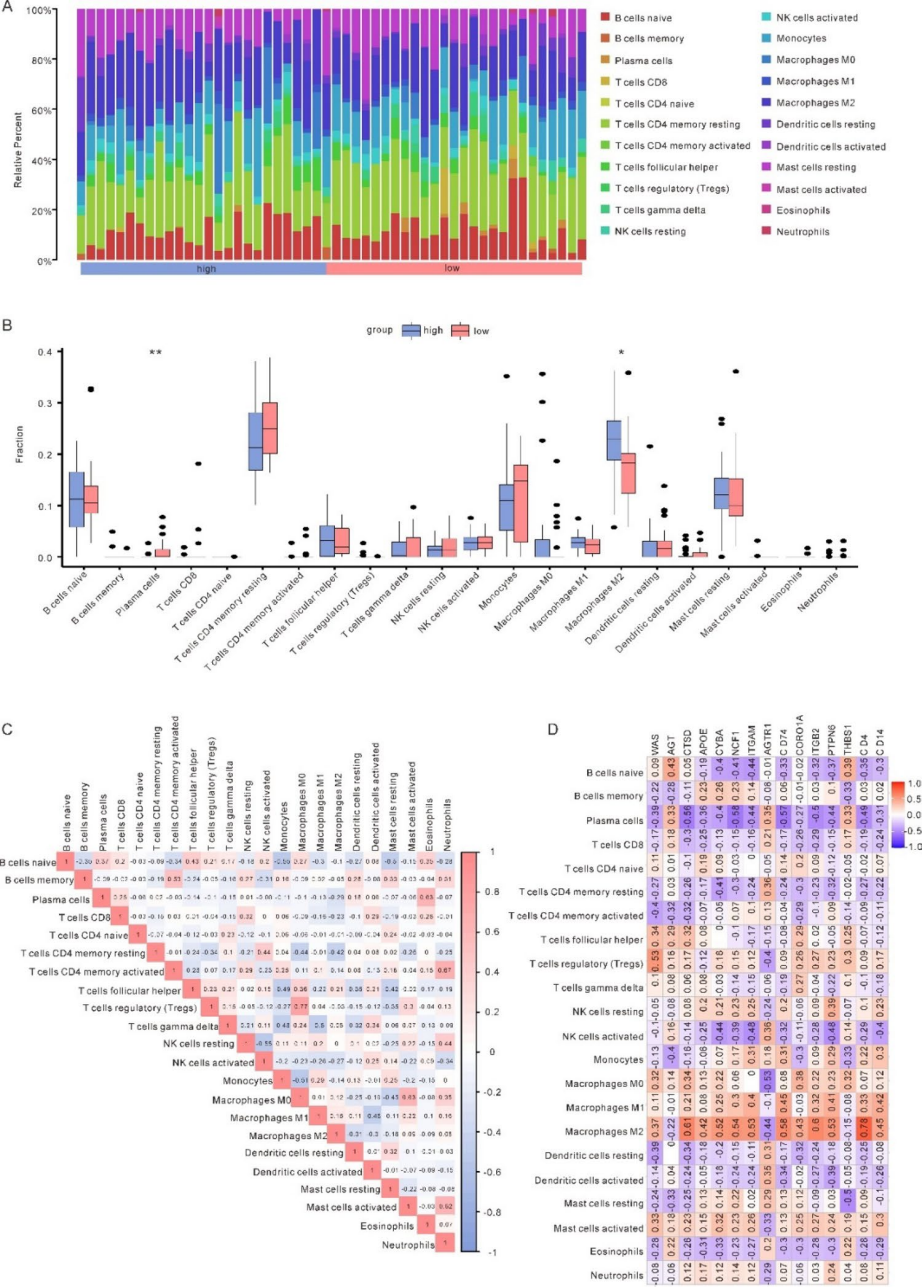
Immune infiltration analysis (**A**) Panorama of infiltration of 22 types of immune cells between high and low OA Pyroptosis score groups. (**B**) Differences of each type of immune cells and immune function between high and Low-Pyroptosis score groups (*p < 0.05; **p < 0.01; ***p < 0.001). (**C**) Correlation heatmap between immune cells. (**D**) Correlation heatmap between immune cells and hub genes. Red color represents positive correlation, purple color represents negative correlation, and the intensity of the color indicates the degree of correlation.

Using CIBERSORT, we analysed the OA datasets’ expression data for 22 different types of immune cell infiltration. After removing all cells with a fraction of 0 in the sample, 22 distinct types of immune cells were able to be identified by their infiltration patterns in each sample. (Fig. 6A). With the Pyroptosis score data in hand, we looked at immune cells that differences in infiltration ratings that were statically important between the two groups. In the two groups, Plasma cells (*p* = 0.0031) and Macrophages M2 (*p* = 0.0123) have a significantly different invasion degree of immune cell and immune function (Fig. 6B). Then we analyzed and visualized the associations between 22 types of immune cells (Fig. 6C). The consequences suggested a different correlation with Macrophages M0 and T cells regulatory (Tregs) (r = 0.77, *p* = 3.35e−11). And the inverse relationship between monocytes and naïve B cells was the strongest of all (r = − 0.55, *p* = 2.5e−05), as well as between NK cells activated and NK cells resting (r = − 0.55, p = 1.99e-05).

Meanwhile, we compared the aforementioned 15 hub genes to 22 M2 macrophages to determine their relationship (Fig. 6D), in which CD4 and macrophages m2 showed the highest positive correlation (r = 0.78, *p* = 7.2e−12). NCF1 was found to have the strongest weak association with plasma cells (r = − 0.58, *p* = 7.62e−06).

### Single cell analysis of chondrocyte subtypes in osteoarthritis and normal samples

**Figure 7:**
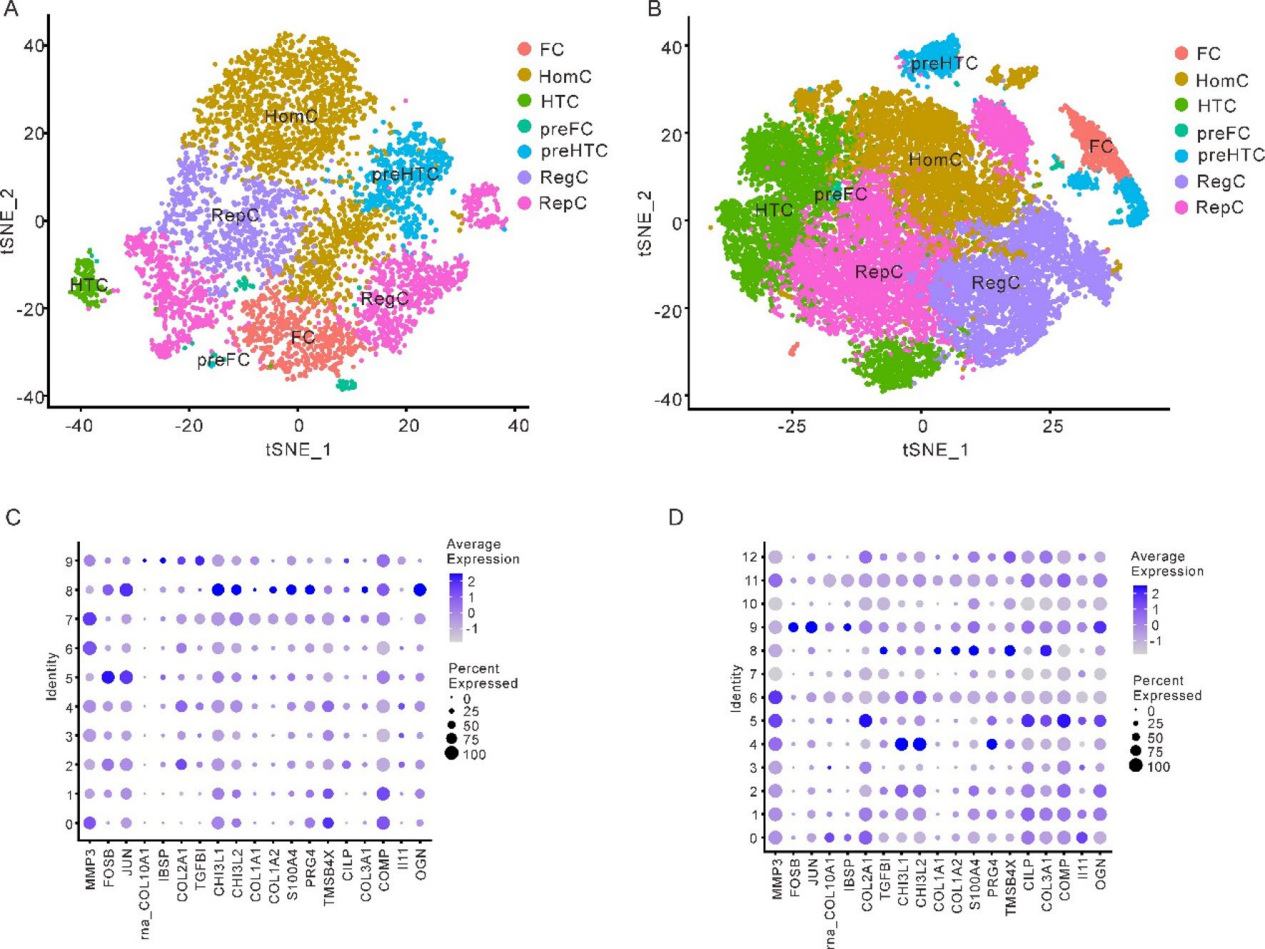
Cell heterogeneity analysis. (**A**) tSNE clusters plots of 7 types of chondrocytes in normal samples. (**B**) tSNE cluster plots of 7 kinds of chondrocytes in OA samples. (**C**) Bubble chart of marker gene expression among different cell clusters in normal samples. (**D**) Bubble chart of marker gene expression among different cell clusters in OA sample. (OA: osteoarthritis).

In the single cell datasets of OA samples and normal samples, the "Seurat" R package was applied to analyze the cell heterogeneity of the single cell data. After removing the cells with mitochondrial gene content greater than 5%, feature quantity < 500 and UMI > 20,000, the cluster plots of 7 chondrocyte subtypes in OA and normal samples were obtained using tSNE clustering (Fig. 7A, 7B). Subsequently, the expression of marker genes in 19 single-cell subgroups in different cell clusters was analyzed, and it could be observed that there were significant differences in marker gene expression among different cell clusters. According to the findings of the study, normal individuals, the MMP3 gene has a low expression in cluster 5 and cluster 8. The IBSP has a low expression in cell cluster 9. COL2A1 and COL3A1 were only highly expressed in cluster 8, while II11 has a low expression in cell cluster 7 (Fig. 7C, Supplementary Fig. S5). For OA samples, cluster 9 showed strong expression of FOSB; COL2A1 was highly expressed in cluster 5. COL1A2 was highly expressed in cluster 8 (Fig. 7D, Supplementary Fig. S6).

### Differential expression and proportions of hub genes in chondrocyte subtypes between osteoarthritis and normal samples

**Figure 8:**
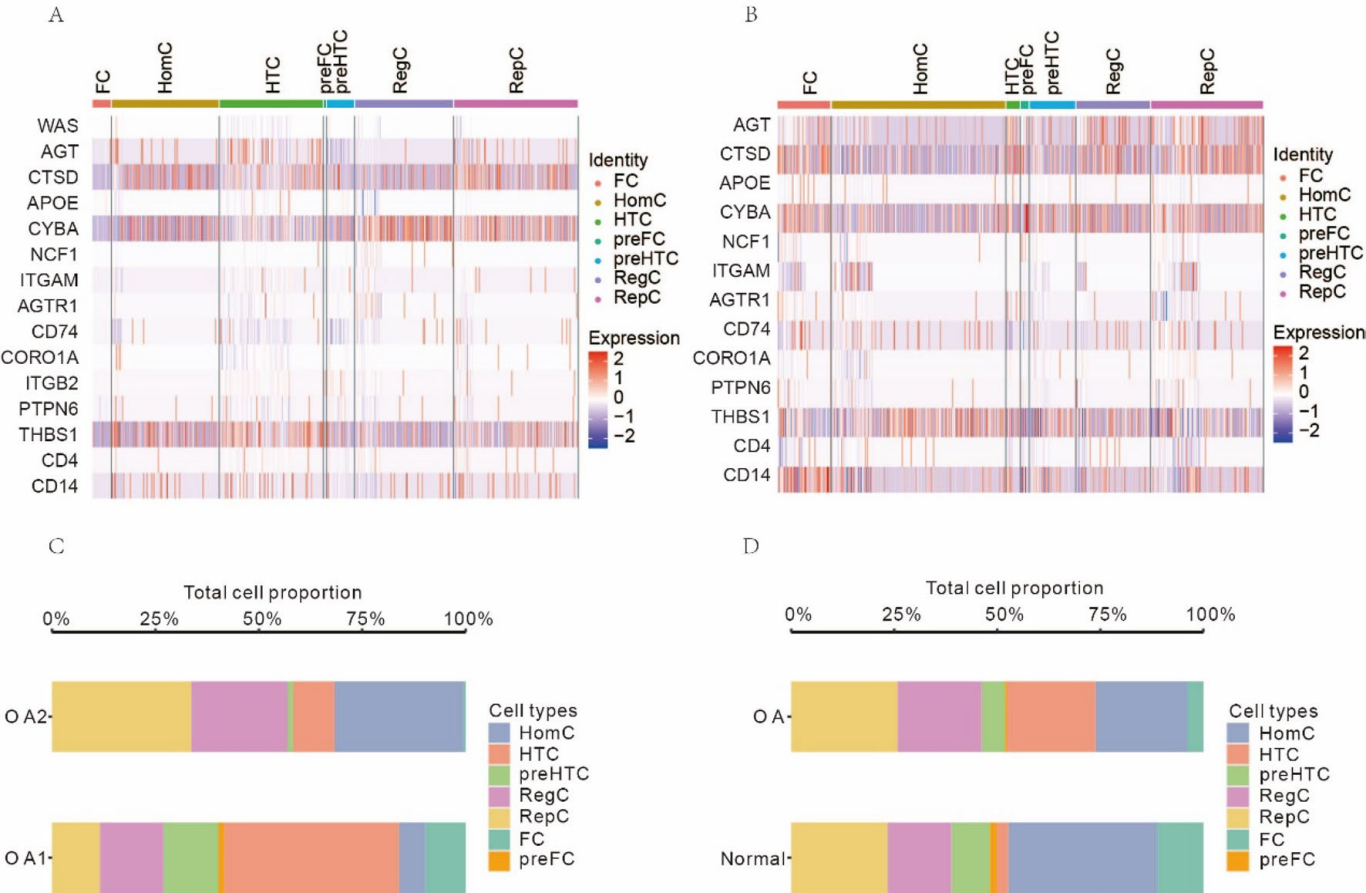
Differential expression analysis of hub genes in different cell populations between OA samples and normal samples, respectively. (**A**) Heatmap of the expression of hub genes in OA samples. (**B**) Heatmap of the expression of hub genes in normal samples. (**C**) The proportion plot of cell groups in the two OA samples. (**D**) Comparison plot of proportion of cell groups in OA samples and normal samples. (OA: osteoarthritis).

The expression of the aforementioned 15 hub genes was analysed differentially among cell populations, and heatmaps depicting the relative abundance of these genes were shown. In OA samples, AGT gene was strongly expressed in HomC cells and HTC cells but little expressed in preHTC cells. HomC cells and RepC cells both have high levels of expression of the gene CTSD. RegC cells showed little expression. The expression of the CYBC gene was high in RegC and RepC cells but low in FC cells (Fig. 8A). In FC cells, expression of the THBS1 gene was rather low. The AGT gene was strongly expressed in RegC cells and weakly expressed in HomC cells in normal samples. FC and Rep cells had strong expression of CTSD, whereas HomC cells had minimal expression. As compared to HomC cells, FC cells had much higher CYBA expression levels. HomC cells showed strong expression of the THBS1 gene, whereas preFC cells showed minimal expression. CD14 was significantly expressed in only FC (Fig. 8B).

Meanwhile, we also analyzed the difference in the proportion of different cell groups between OA patients and normal individuals. We found that, even in OA patients, the proportion of cells derived from different samples varied greatly: HTC cells stood for the highest proportion in OA1 samples, while HomC cells and RepC cells stood for the highest proportion in OA2 samples (Fig. 8C). The ratio of OA sufferers' cells to those of healthy people also varied significantly: HTC cells stood for a relatively high proportion of OA patients, while HTC cells were rarely present in normal samples, and HomC cells were the main cell group (Fig. 8D).

## Discussion

Osteoarthritis (OA) is a prevalent and disabling condition, which poses a significant health and financial burden worldwide. Chondrocyte death-induced cartilage degeneration is a key pathological mechanism in OA. Research has primarily focused on cartilage degeneration and osteophyte formation, synovial fibrosis, characterized by excessive deposition of extracellular matrix, is another pathological change that can cause joint pain and tenderness^44,45^. Fibroblast-like synoviocytes and transforming growth factor-β have been identified as critical players in fibrotic responses^46,47^.

Therefore, understanding the heterogeneity of chondrocytes and the differential expression of pyroptosis-related genes (PRGs) in different cell subgroups could play a vital role in OA progression and treatment, leading to improved diagnosis and therapy. Single-cell RNA sequencing (scRNA-seq) has emerged as a valuable tool in identifying different cell types through transcriptional analysis in various disorders. Studies have used scRNA-seq to define subtypes of chondrocytes with different biological functions, identify transcription programs and major cell populations, screen metabolism-related genes, corresponding transcription factors (TFs), and relevant pathways in the articular chondrocytes of OA patients, and comprehend the molecular characteristics and potential therapeutic targets of chondrocytes in the pathogenesis of OA^48–51^. Despite the benefits of scRNA-seq, few reports have investigated the role of pyroptosis in the pathological process of OA and the heterogeneity of immune cells in OA. In our study, the scRNA-seq analysis of cartilage cells is conducted to explore the heterogeneity and cell-specific gene expression patterns within the chondrocyte population. By examining gene expression at the single-cell level, we can identify subtypes of chondrocytes and investigate how apoptosis-related genes differentially express in these subtypes. This information is highly valuable as it provides insights into specific cell types that may be involved in the pathogenesis of OA and the potential role of cell apoptosis in regulating their functions. When selecting the single-cell RNA-seq dataset, we intentionally chose the chondrocyte samples from GSE169454 to complement the study's investigation of chondrocyte specificity, which may not be fully addressed in the microarray dataset. Through single-cell RNA-seq, we can gain a more in-depth understanding of the specific gene expression patterns and biological processes in chondrocytes during osteoarthritis. This approach contributes to uncovering the critical role of chondrocytes in the development of the disease.

The single-cell analysis provided valuable insights into the heterogeneity of chondrocyte subtypes in OA and normal samples. We identified seven distinct chondrocyte subtypes in OA samples, and these subtypes exhibited differential expression of marker genes associated with cartilage development and matrix remodeling. Notably, the upregulation of MMP3 and COL2A1 in specific chondrocyte clusters in OA samples suggests their potential involvement in cartilage degradation and matrix remodeling processes. These findings are consistent with the known pathophysiology of OA, where increased matrix degradation and altered chondrocyte function play crucial roles in disease progression ^52^.On the other hand, the analysis of the transcriptome dataset allowed us to identify differentially expressed genes (DEGs) associated with pyroptosis and immune response. Pyroptosis is a form of programmed cell death that contributes to the inflammation and tissue damage observed in various diseases, including OA^53^. Our results showed that the expression patterns of pyroptosis-related genes (PRGs) differed significantly between samples with high and low immune scores. These findings suggest that the immune response and pyroptosis may be tightly linked in OA pathogenesis. The identified DEGs are enriched in pathways related to antigen presentation and immune regulation, which further supports the involvement of immune-related processes in OA pathophysiology.

GO enrichment analysis revealed evidence linking DEGs to antigen processing and presentation, which is interesting as human chondrocytes present antigens differently from other cell types^54^. This suggests that "self-antigens" may play a role in the immunopathogenesis of OA when these cells digest aggrecan peptides. Additionally, KEGG analysis showed that DEGs were enriched in Lysosome pathways, which previous studies suggest may mitigate inflammatory chondrocyte cell death and cartilage damage^55^. We also identified pyroptosis-related differentially expressed genes (PRDEGs) using the Wilcoxon rank sum test. We assigned a pyroptosis score to each OA sample using ssGSEA and classified them into either a High-Pyroptosis score group or a Low-Pyroptosis score group using the average pyroptosis index. Univariate and multivariate Cox regression analysis identified 3 PRDEGs (CASP6, NOD1, PYCARD) as independent prognostic factors for OA. While CASP6 has been associated with pyroptosis and tumor prognosis, its role in OA requires further investigation^56^. NOD-1 has been implicated in the chronic and destructive inflammation of the joints in rheumatoid arthritis (RA) patients^57^, but its role in chondrocytes is not fully understood. PYCARD, a component of the inflammasome, may contribute to the clinical symptoms and cartilage breakdown in OA^58^. However, clinical studies on the prognostic significance of CASP6, NOD1, and PYCARD in OA are still lacking.

Afterwards, we found the MEred module was most associated with the Pyroptosis score of each OA patient by WGCNA. And the we identified 15 hub genes (WAS, AGT, CTSD, APOE, CYBA, NCF1, ITGAM, AGTR1, CD74, CORO1A, ITGB2, PTPN6, THBS1, CD4, CD14). We also performed scRNA-seq analysis of cartilage cells and identified multiple subgroups of 7 cell types, including homeostasis chondrocytes (HomC), hypertrophic chondrocytes (HTC), regulatory chondrocytes (RegC), fibrochondrocytes (FC), repair chondrocytes (RepC), and prefibrochondrocytes (preFC). We then performed cell heterogeneity analysis and differential analysis of hub genes in different cell subpopulations. For example, MMP3 reduces the integrity of the joint's non-collagen matrix^59^, while COL2A1, an extracellular signaling molecule found in the cartilage matrix, has the potential to greatly reduce chondrocyte hypertrophy^60^. Abnormal levels of COL1A2 can lead to the production of a matrix associated with mineralization and passivation in OA osteoblasts, and partially promote the production of inflammatory factors^61^. IBSP is related to cartilage development and could promote OA induced by cartilage calcification^62^, while FOSB is a key transcription regulator that could promote bone formation in vivo to increase bone mass^61^. These findings suggest that different cell clusters might be linked to the production of fibrin, collagen, and extracellular matrix, and that cell cluster 9 might be related to chondrocyte development.

Moreover, we conducted the differential analysis of hub genes among different cell subpopulations. AGT was reported to be up-regulated in OA cartilage^63^ and is typically expressed in the traumatic environment caused by cartilage degeneration and inflammation^64^. In our study, AGT had a high expression in HomC and HTC cells, and a low expression in preHTC cells in OA samples. MMP3 and anti-protease SERPINA1 (Alpha-1 anti-trypsin) are primarily produced by HomC and HTC chondrocytes, which are found in abundance in non-invasive cartilage^65^. High expression of HTC cells is related to cartilage degeneration of OA^66^, which might be accompanied by the increase of ACT. CTSD, associated with cartilage development, shows an increase in expression level during the transformation of prochondroblasts into chondroblasts in vivo^67^. Differential expression of AGT and CTSD in Homc cells between OA samples and normal samples might indicate that the development and metabolic disorder of chondrocytes are involved in the progress of OA^23^,which is consistent with our results. THBS1 was found to be upregulated in the synovium of RA and in articular cartilage of OA, and is positively correlated with clinical markers of disease activity/severity, and might play a role in chondrogenesis^68^. Although THBS1 has no direct protective effect on chondrocytes, it might reduce inflammation and be involved in immunomodulatory functions in OA development^69^. Consistent with our observation, these hub genes are engaged in the progression and pathological changes of OA chondrocytes.

In addition, APOE, which primarily participates in cholesterol metabolism and transport, may consequently play a role in the onset of cholesterol-induced osteoarthritis. Variations in cholesterol metabolism exist between different APOE alleles, potentially impacting cholesterol levels and their influence on osteoarthritis^70,71^. Angiotensin II (Ang II), by activating the angiotensin II type 1 receptor (AGTR1), leads to pathological changes in the knee joints of experimental osteoarthritis mice, such as articular cartilage degradation, subchondral bone sclerosis, inflammation, and synovial damage. This highlights the significant role of AGTR1 in the formation of knee joint injuries in experimental osteoarthritis and provides important evidence for further understanding the role of the Ang II/AGTR1 signaling pathway in the pathogenesis of osteoarthritis, as well as the potential for this pathway to serve as a therapeutic target^72^. Felix et al. found variations in CD74 isoform expression were associated with the severity of arthritis in both human patients and experimental models. Additionally, the response to TNF blockade therapy, a common treatment for inflammatory arthritis, was also correlated with CD74 isoform expression. These findings suggest that CD74 may play a crucial role in the pathogenesis of arthritis, and its expression could potentially serve as a biomarker for disease status and treatment response^73^.

As for the results of functional enrichment analysis, reactive oxygen species (ROS) are elevated in OA cartilage and are a major contributor to chronic inflammation. The abnormal Phagosome pathways might contribute to the progression of OA^74^. Enrichment of hub genes was observed in KEGG analyses in Phagosome, Leishmaniasis, Tuberculosis, and other pathways^75^. GSEA and GSVA analysis showed that the oxidative phosphorylation pathway and peroxisome pathway were associated with abnormal chondrocyte metabolism and may be involved in cartilage degeneration and OA progression^76^. PPARs regulate articular cartilage homeostasis and reduce inflammation in human OA cartilage, and their lack may accelerate severe OA by increasing catabolic activity and inhibiting cartilage protection^77^. Therefore, PPARs might be an important target for the treatment of OA. Notch signaling pathway may contribute to enhancing the production of inflammation-related molecules in OA synovial cells and chondrocytes during OA development^78,79^. These findings suggest that targeting these pathways and genes may be a promising approach for developing new OA treatments.

Moreover, we constructed mRNA-miRNA, mRNA-TF, and mRNA-drug networks to investigate potential miRNA, TF, and drug targets of hub genes in the diagnosis and treatment of OA. Building such networks can reveal the mechanisms of miRNA, transcription factors, and drugs in gene regulation, as their interactions with mRNA can lead to the degradation of target mRNA or inhibit its translation, thereby regulating gene expression levels. This helps identify molecularly regulated mRNAs and reveals a complex gene regulatory network. Furthermore, studying these interaction networks allows for a deeper understanding of the roles of miRNA, TFs, and drugs in biological processes such as development, cell differentiation, cell proliferation, and apoptosis, thus promoting comprehension of various physiological and pathological processes within the organism.Secondly, by analyzing the expression changes of miRNAs and TFs in different physiological states or diseases within the network, potential biomarkers can be discovered. The identification of disease-associated miRNAs, TFs, and their target genes facilitates disease diagnosis and treatment. Finally, such interaction networks also aid in the identification of abnormal miRNA and TF expressions that may contribute to the onset and progression of certain diseases, providing potential therapeutic targets for drug development. Therefore, these interaction network analyses offer significant avenues for exploring molecular regulatory mechanisms, biological processes, and disease treatment in bioinformatics research.

We also performed immune infiltration analysis between the High-Pyroptosis score group and the Low-Pyroptosis score group and compared the hub genes to 22 different immune cell subsets to determine their relationship. The results showed that the infiltration degree of plasma cells and macrophages M2 immune cells were significantly different between the two groups, and there is a positive correlation between CD4 and Macrophages M2, while NCF1 has a negative correlation with Plasma cells. Both plasma cells and macrophages are immune cells that are of great importance in the progression of OA and are also important targets of OA immunotherapy^80^. Reprogramming macrophages from the M1 to M2 subtype seems to be an effective treatment option for OA^81^. These findings suggest that targeting these immune cells and pathways may be a promising approach for developing new OA treatments. Further investigation is needed to fully understand the roles of these immune cells and pathways in OA.

Nevertheless, several problems plagued the research. Firstly, it was a retrospective study on the basis of public sequencing data, and the number of OA samples in our study was inadequate. Therefore, how well the OA-related signature we found correlates with prognosis and prediction, as well as the predicted therapeutic targets, should be prospectively verified in large clinical trials. Second, we lack relevant basic experiments to verify the regulatory mechanisms of pyroptosis-related genes in various OA cell populations and the application of predictive prognostic signatures, which more research is planned to investigate. Third, microarray is based on hybridization, while RNA-seq is based on sequencing. Despite applying batch effect correction methods, it is challenging to completely overcome batch effects in microarray datasets. Next, the control (non-OA) samples were not obtained from actual healthy individuals but rather from patients undergoing joint trauma surgery or other non-OA individuals. While this approach may have some limitations, in clinical settings, it is often difficult to obtain knee joint cartilage samples from absolutely healthy individuals due to ethical considerations. Therefore, we chose these samples because they represent the closest available approximation to a “true” non-OA state. Also, the datasets we used are old, and we did not directly compare different tissue types (synovium and cartilage) in this article. Therefore, we have not investigated whether the differential gene expression in different tissues affects the pathogenesis of OA. This analysis would require further investigation in future studies. Despite its limitations, it is believed that to help the large number of untreated OA patients, our research will aid in the development of more effective therapy options and diagnostic biomarkers. The gene expression profile was examined by combining the results of scRNA-seq with batch RNA-seq. These outcomes not only enhance our comprehension of the heterogeneity of OA at the single-cell level, but also provide prognostic related genes based on pyroptosis and the distribution differences of hub genes among different cell groups.

In conclusion, our comprehensive bioinformatics analysis has shed light on the intricate molecular landscape of osteoarthritis (OA) and its potential implications in medicine. We identified key pyroptosis-related genes, including CASP1, CASP3, CASP6, CASP8, GSDMB, NLRP1, NOD1, PLCG1, PRKACA, and PYCARD, which exhibited significant dysregulation in OA samples. These findings highlight the critical role of pyroptosis in OA pathogenesis, providing new insights into the disease’s underlying mechanisms. Furthermore, our study revealed 15 hub genes that could serve as promising biomarkers for predicting OA prognosis. Among them, CD4 and macrophages M2 demonstrated a strong positive correlation, indicating the potential involvement of immune response in OA development and progression. Additionally, our analysis of immune cell infiltration showed distinct patterns of immune cell abundance in OA patients compared to healthy individuals, suggesting the intricate interplay between pyroptosis and the immune system in OA pathophysiology. These results have important implications for medicine, as they offer potential targets for therapeutic interventions aimed atmodulating pyroptosis and immune responses in OA patients. By understanding the molecular basis of OA better, we may develop more precise and effective treatment strategies to halt disease progression, alleviate symptoms, and improve patients' quality of life. However, further experimental validation and clinical studies are necessary to confirm the functional significance of these findings and to translate them into clinical applications. Nevertheless, our study represents a significant step forward in unraveling the complex mechanisms of OA and lays the groundwork for future research and potential personalized treatment options for this debilitating condition.

### Supplementary Information

Supplementary Information.

## Data Availability

The datasets generated during the current study are available in the NCBI (GEO) repository. The datasets generated and analysed during the current study are available in the GEO repository, GSE1428(https://www.ncbi.nlm.nih.gov/geo/query/acc.cgi?acc=GSE1428), GSE8479(https://www.ncbi.nlm.nih.gov/geo/query/acc.cgi?acc=GSE8479).

## References

[1] Lespasio MJ (2017). Knee osteoarthritis: a primer. Perm. J..

[2] Losina E (2015). Lifetime medical costs of knee osteoarthritis management in the United States: Impact of extending indications for total knee arthroplasty. Arthritis Care Res. (Hoboken).

[3] Kolasinski SL (2020). 2019 American College of Rheumatology/arthritis foundation guideline for the management of osteoarthritis of the hand, hip, and knee. Arthritis Care Res. (Hoboken).

[4] Katz JN, Arant KR, Loeser RF (2021). Diagnosis and treatment of hip and knee osteoarthritis: A review. JAMA.

[5] Pas HI (2017). Stem cell injections in knee osteoarthritis: A systematic review of the literature. Br. J. Sports Med..

[6] Fink SL, Cookson BT (2005). Apoptosis, pyroptosis, and necrosis: Mechanistic description of dead and dying eukaryotic cells. Infect. Immun..

[7] Hersh D (1999). The Salmonella invasin SipB induces macrophage apoptosis by binding to caspase-1. Proc. Natl. Acad. Sci..

[8] Man SM, Kanneganti TD (2015). Regulation of inflammasome activation. Immunol. Rev..

[9] An S, Hu H, Li Y, Hu Y (2020). Pyroptosis plays a role in osteoarthritis. Aging Dis..

[10] Mueller BU (2011). Hydroxyurea for children with sickle cell disease: Are we starting too late?. Pediatr. Blood Cancer.

[11] Huber R (2008). Identification of intra-group, inter-individual, and gene-specific variances in mRNA expression profiles in the rheumatoid arthritis synovial membrane. Arthritis Res. Ther..

[12] Woetzel D (2014). Identification of rheumatoid arthritis and osteoarthritis patients by transcriptome-based rule set generation. Arthritis Res. Ther..

[13] Broeren MG (2016). Functional tissue analysis reveals successful cryopreservation of human osteoarthritic synovium. PLoS One.

[14] Guo Y (2017). CD40L-dependent pathway is active at various stages of rheumatoid arthritis disease progression. J. Immunol..

[15] Davis S, Meltzer PSJB (2007). GEOquery: A bridge between the gene expression omnibus (GEO) and BioConductor. Bioinformatics.

[16] Leek JT, Johnson WE, Parker HS, Jaffe AE, Storey JDJB (2012). The sva package for removing batch effects and other unwanted variation in high-throughput experiments. Bioinformatics.

[17] Ritchie ME (2015). limma powers differential expression analyses for RNA-sequencing and microarray studies. Nucleic Acids Res..

[18] Satija R, Farrell JA, Gennert D, Schier AF, Regev A (2015). Spatial reconstruction of single-cell gene expression data. Nat. Biotechnol..

[19] Kim S, Kang D, Huo Z, Park Y, Tseng GCJB (2018). Meta-analytic principal component analysis in integrative omics application. Bioinformatics.

[20] Ye Y, Dai Q, Qi H (2021). A novel defined pyroptosis-related gene signature for predicting the prognosis of ovarian cancer. Cell Death Discov..

[21] Chou C-H (2020). Synovial cell cross-talk with cartilage plays a major role in the pathogenesis of osteoarthritis. Sci. Rep..

[22] Korsunsky I (2019). Fast, sensitive and accurate integration of single-cell data with Harmony. Nat. Methods.

[23] Ji Q (2019). Single-cell RNA-seq analysis reveals the progression of human osteoarthritis. Ann. Rheum. Dis..

[24] Yoshihara K (2013). Inferring tumour purity and stromal and immune cell admixture from expression data. Nat. Commun..

[25] Villanueva, R. A. M. & Chen, Z. J. (Taylor & Francis, 2019).

[26] Kolde, R. *pheatmap: Pretty Heatmaps* (2015).

[27] Hänzelmann S, Castelo R, Guinney JJBB (2013). GSVA: Gene set variation analysis for microarray and RNA-seq data. BMC Bioinform..

[28] Subramanian A (2005). Gene set enrichment analysis: A knowledge-based approach for interpreting genome-wide expression profiles. Proc. Nat. Acad. Sci..

[29] Wu, T. *et al.* (2021).

[30] Liberzon, A. *et al.* Molecular signatures database (MSigDB) 3.0. **27**, 1739–1740 (2011). 10.1093/bioinformatics/btr260 PMC3106198 21546393

[31] Langfelder P, Horvath SJBB, Langfelder P, Horvath S (2009). WGCNA: An R package for weighted correlation network analysis. BMC Bioinform..

[32] Szklarczyk D (2019). STRING v11: Protein–protein association networks with increased coverage, supporting functional discovery in genome-wide experimental datasets. Nucleic Acids Res..

[33] Shannon P (2003). Cytoscape: A software environment for integrated models of biomolecular interaction networks. Genome Res..

[34] Chin C-H (2014). cytoHubba: Identifying hub objects and sub-networks from complex interactome. BMC Syst. Biol..

[35] Yu G (2020). Gene ontology semantic similarity analysis using GOSemSim. Methods Mol. Biol..

[36] Ogata H (1999). KEGG: Kyoto encyclopedia of genes and genomes. Nucleic Acids Res..

[37] Li JH, Liu S, Zhou H, Qu LH, Yang JH (2014). starBase v2.0: Decoding miRNA-ceRNA, miRNA-ncRNA and protein-RNA interaction networks from large-scale CLIP-Seq data. Nucleic Acids Res..

[38] Chen Y, Wang XJN (2020). miRDB: An online database for prediction of functional microRNA targets. Nucleic Acids Res..

[39] Zhou, K.-R. *et al.* ChIPBase v2. 0: decoding transcriptional regulatory networks of non-coding RNAs and protein-coding genes from ChIP-seq data. gkw965 (2016). 10.1093/nar/gkw965 PMC5210649 27924033

[40] Zhang Q (2020). hTFtarget: A comprehensive database for regulations of human transcription factors and their targets. Genom. Proteomics Bioinform..

[41] Davis, A. P. *et al.* Comparative toxicogenomics database (CTD): update 2021. **49**, D1138–D1143 (2021). 10.1093/nar/gkaa891 PMC7779006 33068428

[42] Chen B, Khodadoust MS, Liu CL, Newman AM, Alizadeh AA (2018). Cancer Systems Biology.

[43] Harrell FEJB (2017). Regression modeling strategies. Bios.

[44] Suri S, Walsh DA (2012). Osteochondral alterations in osteoarthritis. Bone.

[45] Chen D (2017). Osteoarthritis: Toward a comprehensive understanding of pathological mechanism. Bone Res..

[46] Han D (2020). The emerging role of fibroblast-like synoviocytes-mediated synovitis in osteoarthritis: an update. J. Cell Mol. Med..

[47] Zhang L (2019). Increased HIF-1α in knee osteoarthritis aggravate synovial fibrosis via fibroblast-like synoviocyte pyroptosis. Oxid. Med. Cell Longev..

[48] Cao F (2022). Identification of the OA-related metabolism-related genes, corresponding transcription factors, relevant pathways, and specific bioactive small molecules. Int. Immunopharmacol..

[49] Hu X (2022). Identification of cellular heterogeneity and immunogenicity of chondrocytes via single-cell RNA sequencing technique in human osteoarthritis. Front. Pharmacol..

[50] Wang X (2021). Comparison of the major cell populations among osteoarthritis, Kashin–Beck disease and healthy chondrocytes by single-cell RNA-seq analysis. Cell Death Dis..

[51] Lv Z (2022). Single cell RNA-seq analysis identifies ferroptotic chondrocyte cluster and reveals TRPV1 as an anti-ferroptotic target in osteoarthritis. EBioMedicine.

[52] Goldring MB, Goldring SR (2010). Articular cartilage and subchondral bone in the pathogenesis of osteoarthritis. Ann. N. Y. Acad. Sci..

[53] McAuley JL (2013). Activation of the NLRP3 inflammasome by IAV virulence protein PB1-F2 contributes to severe pathophysiology and disease. PLoS Pathog..

[54] Sengprasert P (2022). Upregulation of antigen presentation function and inflammation in chondrocytes by induction of proteoglycan aggrecan peptides (P16-31 and P263-280). Clin. Exp. Rheumatol..

[55] Na HS (2021). Metformin attenuates monosodium-iodoacetate-induced osteoarthritis via regulation of pain mediators and the autophagy–lysosomal pathway. Cells.

[56] Guo K (2022). CASP6 predicts poor prognosis in glioma and correlates with tumor immune microenvironment. Front. Oncol..

[57] Yokota K (2012). The pattern-recognition receptor nucleotide-binding oligomerization domain–containing protein 1 promotes production of inflammatory mediators in rheumatoid arthritis synovial fibroblasts. Arthritis Rheum..

[58] Zhang B (2020). SQSTM1-dependent autophagic degradation of PKM2 inhibits the production of mature IL1B/IL-1β and contributes to LIPUS-mediated anti-inflammatory effect. Autophagy.

[59] Burrage PS, Mix KS, Brinckerhoff CE (2006). Matrix metalloproteinases: Role in arthritis. Front. Biosci..

[60] Lian C (2019). Collagen type II suppresses articular chondrocyte hypertrophy and osteoarthritis progression by promoting integrin β1-SMAD1 interaction. Bone Res..

[61] Couchourel D (2009). Altered mineralization of human osteoarthritic osteoblasts is attributable to abnormal type I collagen production. Arthritis Rheum..

[62] Jiang S, Zhang C, Lu Y, Yuan F (2022). The molecular mechanism research of cartilage calcification induced by osteoarthritis. Bioengineered.

[63] Wang W (2020). AGT, targeted by miR-149-5p, promotes IL-6-induced inflammatory responses of chondrocytes in osteoarthritis via activating JAK2/STAT3 pathway. Clin. Exp. Rheumatol..

[64] Li Z (2020). The tissue-renin-angiotensin-system of the human intervertebral disc. Eur. Cell Mater..

[65] Chou CH (2020). Synovial cell cross-talk with cartilage plays a major role in the pathogenesis of osteoarthritis. Sci. Rep..

[66] Rim YA, Nam Y, Ju JH (2020). The role of chondrocyte hypertrophy and senescence in osteoarthritis initiation and progression. Int. J. Mol. Sci..

[67] Ramesova A (2022). Autophagy-related proteases accompany the transition of pre-chondrogenic cells into chondroblasts. Ann. Anat..

[68] Maumus M (2017). Thrombospondin-1 partly mediates the cartilage protective effect of adipose-derived mesenchymal stem cells in osteoarthritis. Front. Immunol..

[69] Robb KP, Audet J, Gandhi R, Viswanathan S (2022). Putative critical quality attribute matrix identifies mesenchymal stromal cells with potent immunomodulatory and angiogenic "fitness" ranges in response to culture process parameters. Front. Immunol..

[70] Gierman LM (2014). Osteoarthritis development is induced by increased dietary cholesterol and can be inhibited by atorvastatin in APOE*3.LeidenCETP mice–a translational model for atherosclerosis. Ann. Rheum. Dis..

[71] Farnaghi S (2017). Protective effects of mitochondria-targeted antioxidants and statins on cholesterol-induced osteoarthritis. FASEB J..

[72] de Sá GA (2021). Angiotensin II triggers knee joint lesions in experimental osteoarthritis. Bone.

[73] Clanchy FIL (2022). Disease status in human and experimental arthritis, and response to TNF blockade, is associated with MHC class II invariant chain (CD74) isoform expression. J. Autoimmun..

[74] Ansari MY, Ahmad N, Haqqi TM (2020). Oxidative stress and inflammation in osteoarthritis pathogenesis: Role of polyphenols. Biomed. Pharmacother..

[75] Gao N (2020). Metabonomic-transcriptome integration analysis on osteoarthritis and rheumatoid arthritis. Int. J. Genom..

[76] Zheng L, Zhang Z, Sheng P, Mobasheri A (2021). The role of metabolism in chondrocyte dysfunction and the progression of osteoarthritis. Ageing Res. Rev..

[77] Huang G (2021). Role of peroxisome proliferator-activated receptors in osteoarthritis (Review). Mol. Med. Rep..

[78] Hilton MJ (2008). Notch signaling maintains bone marrow mesenchymal progenitors by suppressing osteoblast differentiation. Nat. Med..

[79] Saito T, Tanaka S (2017). Molecular mechanisms underlying osteoarthritis development: Notch and NF-κB. Arthritis Res. Ther..

[80] Dolzani P, Manferdini C, Meliconi R, Lisignoli G, Pulsatelli L (2022). Preliminary study on immune cells in the synovium of end-stage osteoarthritis and rheumatoid arthritis patients: Neutrophils and IgG4-secreting plasma cells as differential diagnosis candidates. Acta Histochem..

[81] Zhang H, Cai D, Bai X (2020). Macrophages regulate the progression of osteoarthritis. Osteoarthr. Cartil..

