# Supplementary material for: Integrated single-cell and bulk RNA sequencing analysis identified pyroptosis-related signature for diagnosis and prognosis in osteoarthritis
Source: Sci Rep. 2023 Oct 18;13:17757. doi: 10.1038/s41598-023-44724-0 (PMC10584952; doi:10.1038/s41598-023-44724-0)
Supplement: Supplementary file 1 — Supplementary Information. [file 41598_2023_44724_MOESM1_ESM.pdf]

Supplementary information

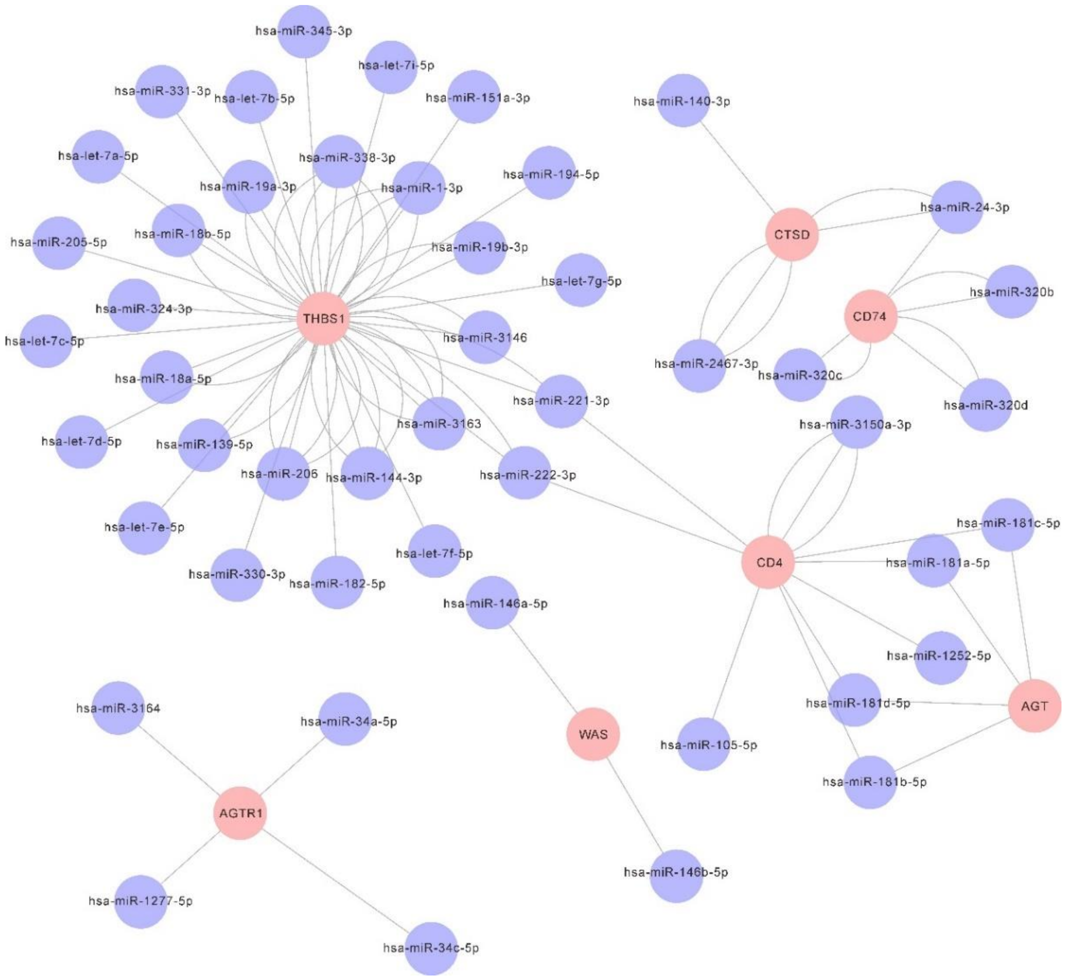

Supplementary Fig. S1. Construction of mRNA-miRNA interaction network.

The mRNA-miRNA interaction network of hub genes, the red oval block is mRNA, the blue oval block is miRNA.

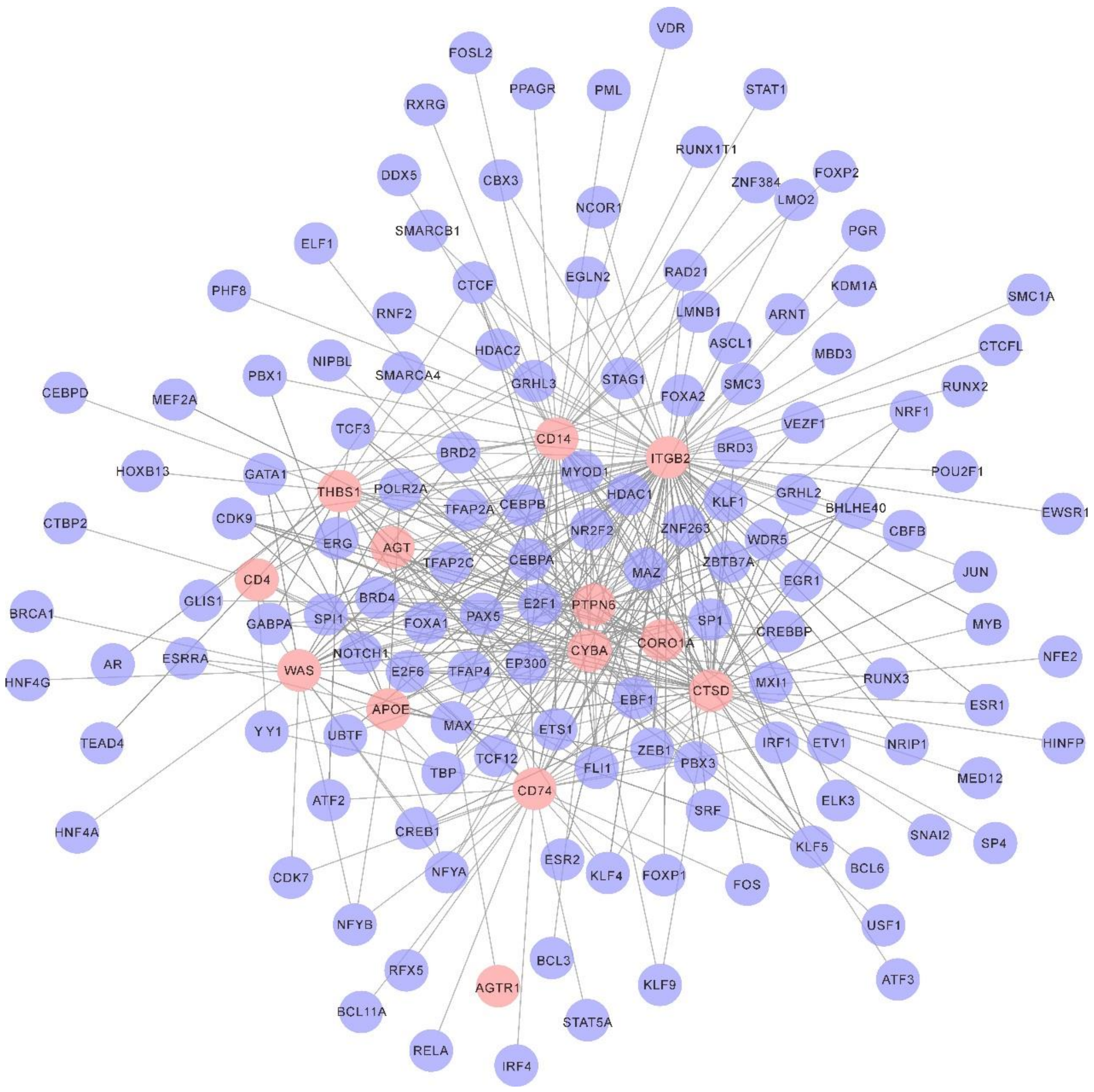

Supplementary Fig. S2. Construction of mRNA-TF interaction network. The mRNA-TF interaction network of hub genes, the red oval block is mRNA, the blue oval block is TF. (TF: Transcription factors)

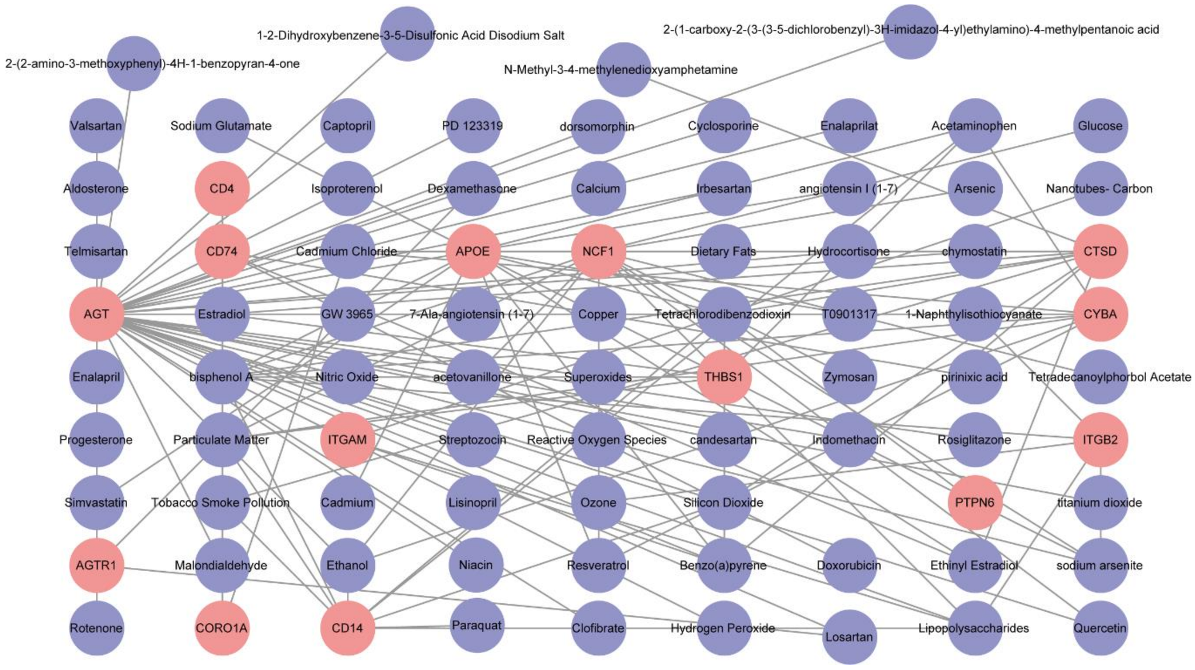

Supplementary Fig. S3. Construction of mRNA--drugs interaction network.

The mRNA--drugs interaction network of hub genes, the red oval block is mRNA, the blue oval block is drugs.

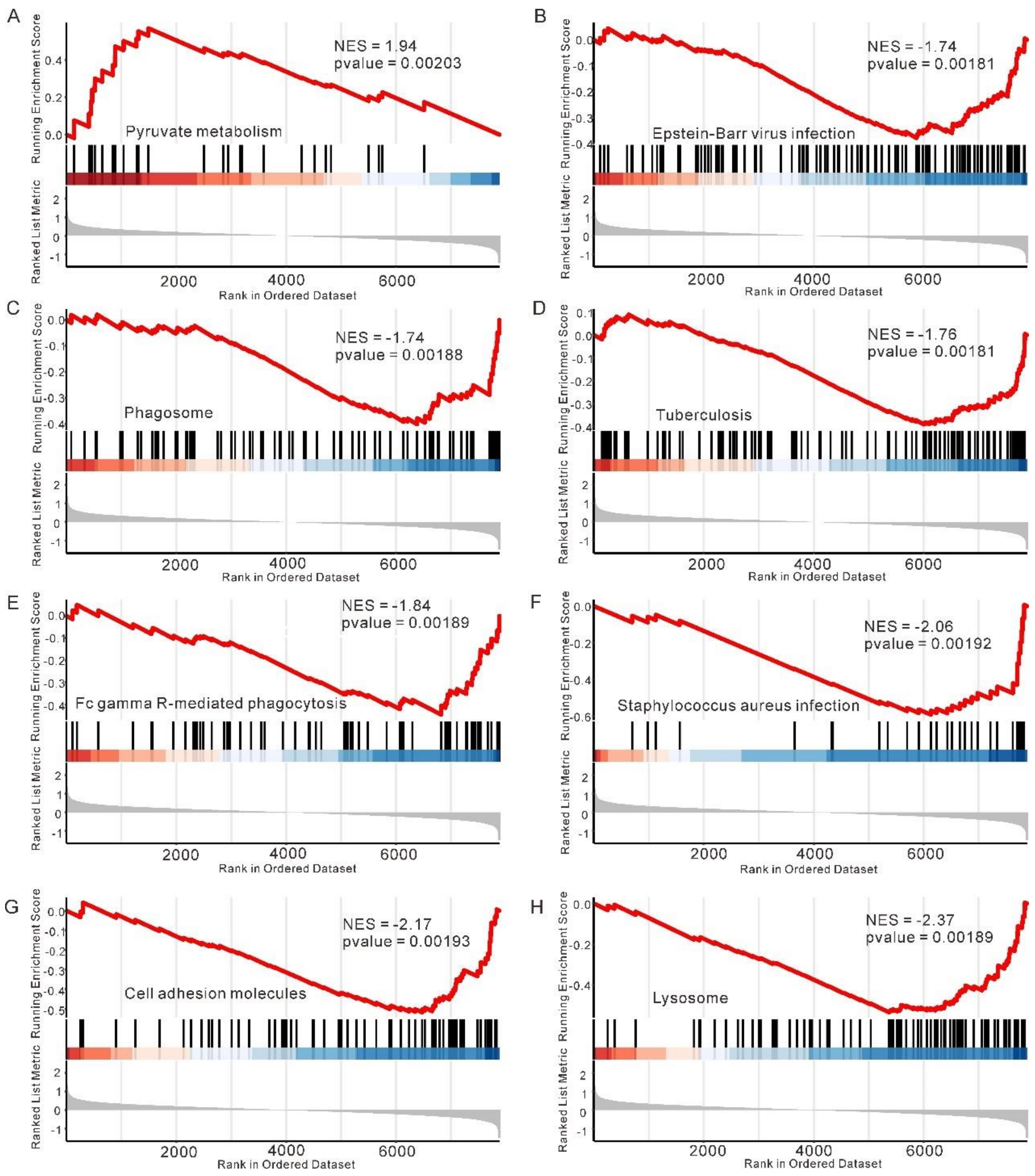

Supplementary Fig. S4. GSEA between High-Pyroptosis score group and Low-Pyroptosis score group. (A) Specific enrichment of the Pyruvate metabolism pathway. (B: Specific enrichment of Epstein-Barr virus infection pathway. C: Specific enrichment of Phagosome pathway. D: Tuberculosis pathway enrichment. E: Enrichment of Fc gamma R-mediated phagocytosis pathway. F: Specific enrichment of Staphylococcus aureus infection pathway. G: Enrichment of Cell adhesion molecules pathway. H: Specific enrichment of Lysosome pathway. (GSEA: Gene set enrichment analysis).

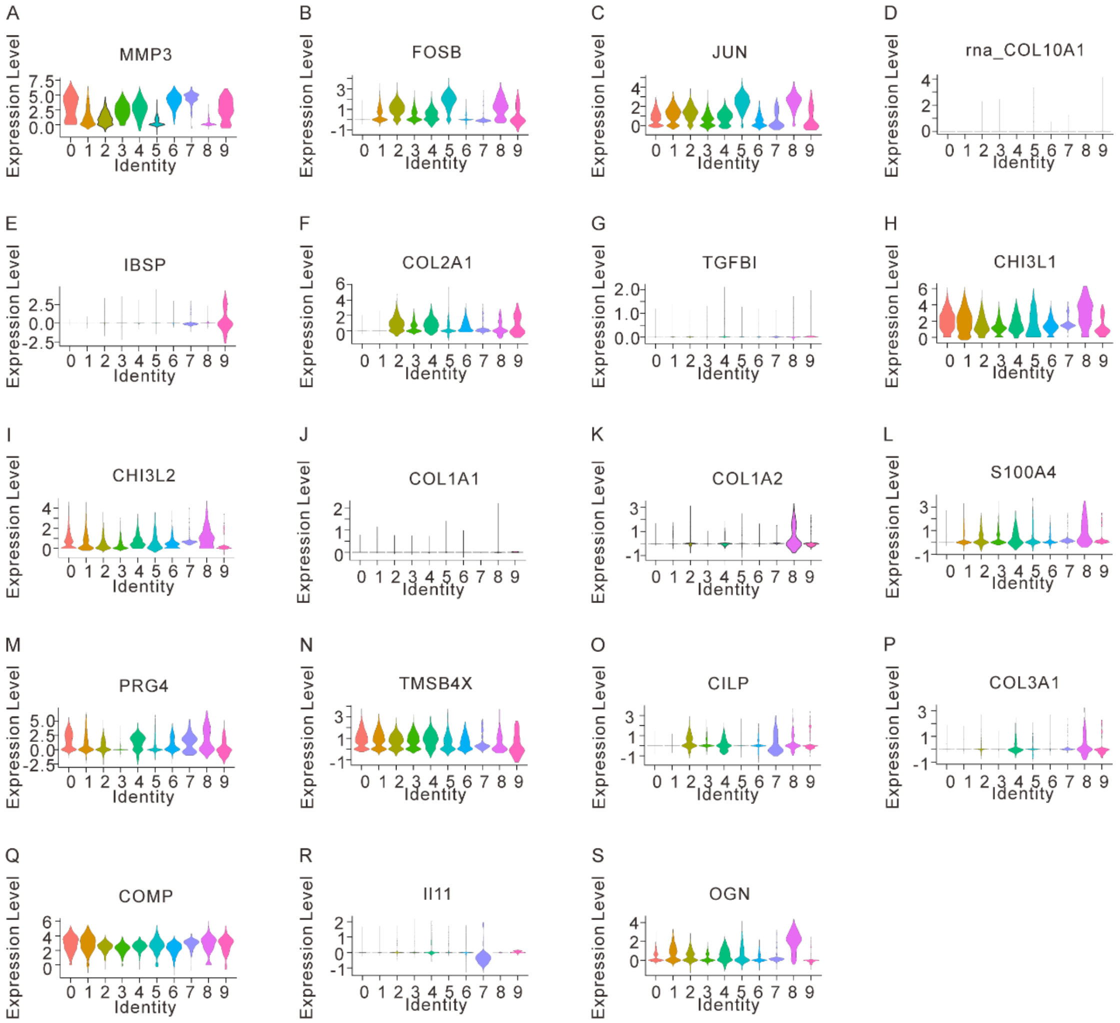

Supplementary Fig. S5. The expression of marker genes in 19 single-cell subgroups in different cell clusters of normal individuals.

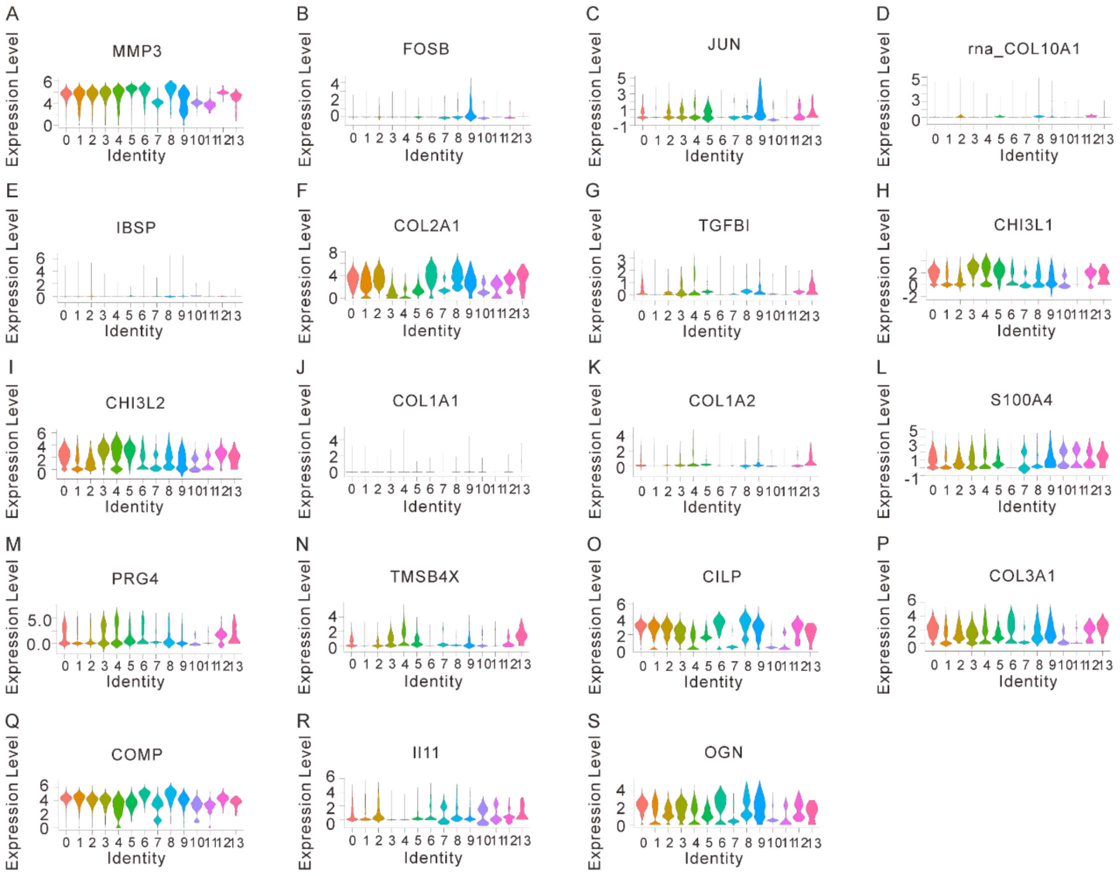

Supplementary Fig. S6. The expression of marker genes in 19 single-cell subgroups in different cell clusters of normal individuals.

Supplementary Table S1. A total of 238 genes containing in the MEred module contains

Gene name
DOK3, ADAP2, TNFAIP2, COLGALT1, PSTPIP1, NCF1, ZSWIM8, IRF5, CYBA, PRR16, WAS, PYCARD, NFYB, SELPLG, LAMA2, MAN2B1, ARRB2, FZD6, AGTR1, PIK3CD, LGALS9, INPP4B, IGFLR1, PLCB4, DENND4B, TMEM47, ITGAM, RHOBTB3, VASP, ARHGAP22, ACOT11, STXBP2, COTL1, CDH19, LOX, ESPL1, STX10, NEDD9, CNN3, TBC1D2, TSPO, ADCY7, ABCC3, RGS19, TSPAN15, SLC15A3, ABCC5, GADD45A, CDC42EP3, CORO1A, PERP, UNC93B1, CD300C, ANPEP, SLC23A2, FGF18, MECOM, CALCRL, GALNT10, GIPC2, PLA2G15, GMIP, CSRP2, SEPT11, ITGB2, SMG9, MARK2, CYP7B1, SLC22A18, SLC2A6, C2, MYB, GRN, NAGA, SERPINA5, RUNDC3B, SNED1, TPCN1, RASSF4, DAPK1, SYT17, TACSTD2, TNFSF13, ADAP1, NAGK, CLN6, HOXB6, PTPN6, TXNRD2, SLC6A12, OGDH, ZDHHC24, ABCC9, SIRPA, TIAM1, KIAA0513, DSG2, GAA, PLK2, KL, CTSD, WASF3, TRPV2, SYNE2, DLX4, THEMIS2, ARHGAP29, S1PR1, RAPGEF5, ATP6V0E2, CD4, EMCN, PDGFRL, ABLIM1, ASPN, GAB1, SCRN1, GPM6B, SPOCK1, PELI2, EHF, ERG, SLCO2A1, MCOLN1, CILP, SLC1A4, RNF144A, KLF9, DOCK9, EPHB2, DSP, DPP4, TRIM14, ANK2, S100A3, SGCE, PTPRB, CLU, PTGS1, RNASET2, PREX2, STRA6, PLTP, HSD17B14, CD14, SEMA5A, CD74, TMEM51, AJAP1, POSTN, GNG11, TUBB2B, RBMS3, SRPX, TSPAN13, TM4SF1, SVEP1, PTPRK, OSBPL10, KCNN3, SOCS2, C1orf21, SORL1, MAFF, SYNGR3, NR1H3, STEAP1B, FILIP1L, KCNE4, DIO2, STX1A, KLHL3, SPINT1, THBS1, MPDZ, RMDN3, PODXL, SIX3, AP1B1, CD200, ABCB1, FLT1, DOPEY2, RPGR, SIPA1L1, SMCO4, MTUS2, LRRN3, ARHGEF28, ADAMTS9, MYO5C, CLIC5, ANGPT2, RTN1, SMAGP, SFRP1, RPP25, FBN1, CNNM4, ITM2A, CDK20, GRB10, CXCL14, TMOD2, APOE, TSPAN2, ID4, PAQR4, PTGER3, RPS6KA1, SIL1, ADAM12, MAPK13, KDR, LAMB1, YWHAH, MMP16, ZMYND10, EFNA1, FZD4, COL21A1, CA8, CHN1, CRISPLD2, PDE5A, SUSD4, CARD14, DNASE2, BTC, NID1, TWIST1, BACE2, ALDH2, AGT, APOLD1, NFKBIE, DCLK1, MAP4K4

Supplementary Table S2. The104 key genes

Gene name
ARRB2, NCF1, TNFSF13, CYBA, PLA2G15, SELPLG, ABCC3, DSG2, COTL1, AP1B1, CD14, PTPN6, NAGA, NR1H3, TRIM14, TRPV2, CD74, CD4, ID4, PYCARD, ITGAM, RNASET2, GRN, TBC1D2, IRF5, ANGPT2, ADAP2, C2, TMEM51, NID1, TMEM47, UNC93B1, ITGB2, MPDZ, SYNE2, CD300C, CDC42EP3, TSPAN15, CORO1A, ERG, LGALS9, TSPO, PRR16, RPS6KA1, GRB10, SYT17, DAPK1, CSRP2, STXBP2, SORL1, APOE, C1orf21, PSTPIP1, MCOLN1, ADCY7, SLC2A6, IGFLR1, FLT1, MAN2B1, LAMA2, GPM6B, SLC15A3, RNF144A, MMP16, APOLD1, COLGALT1, LRRN3, THEMIS2, CTSD, PTGS1, WASF3, FZD6, AGT, DNASE2, BTC, CNN3, PTGER3, SMCO4, INPP4B, GAB1, SOCS2, RAPGEF5, KL, WAS, SCRN1, SLC6A12, DOK3, THBS1, MAPK13, ARHGAP29, ADAMTS9, MECOM, TNFAIP2, PLK2, AGTR1, MAP4K4, ABCC9, ZMYND10, HOXB6, SUSD4, PERP, PLCB4, EHF, SVEP1
